# Assessing the Effect of Electronic Health Record Data Quality on Identifying Patients With Type 2 Diabetes: Cross-Sectional Study

**DOI:** 10.2196/56734

**Published:** 2024-08-27

**Authors:** Priyanka Dua Sood, Star Liu, Harold Lehmann, Hadi Kharrazi

**Affiliations:** 1 Bloomberg School of Public Health, Johns Hopkins University, 615 N Wolfe St, Baltimore, MD, 21205, United States, 1 443-287-8264; 2 School of Medicine, Johns Hopkins University, Baltimore, MD, United States

**Keywords:** electronic health record, EHR, EHRs, record, records, computable, phenotyping, phenotype, phenotypes, computable phenotypes, data quality, data science, chronic, identify, identification, data types—diagnosis data, medication data, laboratory data, type-2 diabetes, diabetes, diabetic, DM, type 2, hospital system, clinical research and trial, diagnosis, diagnoses, diagnose, diagnostic, diagnostics, phenotypic

## Abstract

**Background:**

Increasing and substantial reliance on electronic health records (EHRs) and data types (ie, diagnosis, medication, and laboratory data) demands assessment of their data quality as a fundamental approach, especially since there is a need to identify appropriate denominator populations with chronic conditions, such as type 2 diabetes (T2D), using commonly available computable phenotype definitions (ie, phenotypes).

**Objective:**

To bridge this gap, our study aims to assess how issues of EHR data quality and variations and robustness (or lack thereof) in phenotypes may have potential impacts in identifying denominator populations.

**Methods:**

Approximately 208,000 patients with T2D were included in our study, which used retrospective EHR data from the Johns Hopkins Medical Institution (JHMI) during 2017‐2019. Our assessment included 4 published phenotypes and 1 definition from a panel of experts at Hopkins. We conducted descriptive analyses of demographics (ie, age, sex, race, and ethnicity), use of health care (inpatient and emergency room visits), and the average Charlson Comorbidity Index score of each phenotype. We then used different methods to induce or simulate data quality issues of completeness, accuracy, and timeliness separately across each phenotype. For induced data incompleteness, our model randomly *dropped* diagnosis, medication, and laboratory codes independently at increments of 10%; for induced data inaccuracy, our model randomly *replaced* a diagnosis or medication code with another code of the same data type and induced 2% incremental change from −100% to +10% in laboratory result values; and lastly, for timeliness, data were modeled for induced *incremental shift* of date records by 30 days to 365 days.

**Results:**

Less than a quarter (n=47,326, 23%) of the population overlapped across all phenotypes using EHRs. The population identified by each phenotype varied across all combinations of data types. Induced incompleteness identified fewer patients with each increment; for example, at 100% diagnostic incompleteness, the Chronic Conditions Data Warehouse phenotype identified zero patients, as its phenotypic characteristics included only diagnosis codes. Induced inaccuracy and timeliness similarly demonstrated variations in performance of each phenotype, therefore resulting in fewer patients being identified with each incremental change.

**Conclusions:**

We used EHR data with diagnosis, medication, and laboratory data types from a large tertiary hospital system to understand T2D phenotypic differences and performance. We used induced data quality methods to learn how data quality issues may impact identification of the denominator populations upon which clinical (eg, clinical research and trials, population health evaluations) and financial or operational decisions are made. The novel results from our study may inform future approaches to shaping a common T2D computable phenotype definition that can be applied to clinical informatics, managing chronic conditions, and additional industry-wide efforts in health care.

## Introduction

Type 2 diabetes (T2D) is a common chronic disease affecting more than 11% of the US population [1]. Given the rapidly increasing burden of T2D on health care services and resources, health systems have devised strategies to address such demands by identifying and managing T2D patients across their populations [1]. However, despite clinical guidelines to identify T2D at the point of care, identifying T2D patients in large clinical repositories is still a challenge [2]. Ambiguity of algorithms to identify T2D patients in complex data sets and data quality issues in routine clinical data sources such as electronic health records (EHRs) are still hindering the development of generalizable approaches to identify T2D patients across different health systems.

Over the last two decades, several approaches have been developed for health care professionals to identify patients with T2D in large clinical data repositories. Multiple T2D phenotype definitions (also known as computational algorithms) are available that define characteristics to identify T2D populations. Some examples of T2D definitions include the Surveillance, Prevention, and Management of Diabetes Mellitus (SUPREME-DM) [3], the Centers for Medicare and Medicaid Services (CMS) Chronic Conditions Data Warehouse (CCW) [4], the Electronic Medical Records and Genomics (eMERGE) Northwestern Group [5], and the Durham Diabetes Coalition (DDC) [6] phenotypes. Each phenotype definition has its own set of inclusion and exclusion criteria using different data types. Data types may include *International Statistical Classification of Diseases*, *Tenth Edition* (*ICD-10*) diagnosis codes, RxNorm medication codes, and Logical Observation Identifiers Names and Codes (LOINC) laboratory codes, with sequences and frequencies of occurrences of diagnosis, medication and laboratory codes; defined time periods; and care pathways. However, despite these detailed definitions, it is unclear which of the existing T2D phenotypes are prone to inherit data quality issues from clinical data sources such as EHRs. The uncertainty of T2D phenotypes’ performance for identifying populations with T2D using real-world data has led to the lack of a universally agreed-upon T2D phenotype.

The concept of data quality in health care varies based on the problems and functional needs of end users. Health care providers, data scientists, or policy makers may differ in their approach to data quality and its significance in practice [7]. Typically, health care data such as captured in EHRs are collected for continuity of clinical care, coding, and billing purposes, and not necessarily to answer specific research questions. Thus, the quality of data collected in EHRs may be sufficient for clinical purposes but may not meet the needs of a researcher or population health intervention. For example, a clinician may identify a patient with T2D at the point of care despite having incomplete data; however, a T2D phenotyping algorithm may miss the same patient in a large EHR data warehouse due to the underlying data quality issues; hence, the patient may inadvertently be excluded from a research study or population health intervention.

Various data quality frameworks have been proposed to measure the quality of health care data. Completeness, accuracy, and timeliness are a few key data quality characteristics that are used across several frameworks [7-9]. The assessment for completeness defines how complete the data are, what the missing elements are, and how usable the data are in their “as is” format. Accuracy is the correctness and consistency of the data elements [8], and timeliness is how recent or current the data are for research and analysis. The assessment of data quality in health care is crucial with the continuous and prominent use of EHRs; however, despite the increasing use of EHR data to identify patients with T2D, the effect of varying levels of key data quality characteristics (ie, completeness, accuracy, and timeliness) on T2D phenotypes is still unknown.

The ongoing challenge of understanding the effect of key data quality issues on T2D phenotypes is further exacerbated by the fact that T2D phenotypes use multiple data types, such as diagnosis codes, medications, and laboratory results. Additionally, given the variability of key data quality issues of these data types across EHRs [10,11], measuring the effect of key existing data quality issues on T2D phenotypes in one EHR may not translate into generalizable findings. For example, one provider’s EHR may suffer from incompleteness of diagnosis codes, while another provider’s EHR may be affected by inaccurate medication data. Thus, to measure the effect of an EHR’s data quality on T2D phenotypes, varying (simulated) levels of key data quality characteristics across all data types (ie, diagnosis, medication, and laboratory) used by T2D phenotypes should be studied. These simulated levels of key data quality issues will in turn help providers to compare their EHR data quality issues with the simulated levels, contrast the potential impact of such data quality issues on identifying T2D patients using various phenotypes, and eventually select the most suitable T2D phenotype for their EHR data.

Currently, evidence is lacking on the effect of data quality issues (eg, completeness, accuracy, and timeliness) and the identification of T2D populations in large clinical data sources such as EHRs. This gap in evidence is further amplified given the variations in characteristics of published T2D phenotypes and potential discrepancies in underlying data types (ie, diagnosis, medication, and laboratory) in EHRs. To address these gaps, our study aimed to assess the impact of varying (simulated) levels of data quality issues across the different data types used by T2D phenotypes. Our study findings can inform health care providers and other stakeholders to select T2D phenotype algorithms that best match their underlying EHR data quality issues.

## Methods

### Data Source

Our cross-sectional study used retrospective EHR data from the Johns Hopkins Medical Institute (JHMI) data warehouse over a 3-year period from 2017 to 2019. Clinical data included T2D primary diagnostic data (*ICD-10*), laboratory data (LOINC), and medication data (RxNorm). The demographic data included age, sex, race, ethnicity, and the patients’ residential state.

### Study Population

Our overall study population included approximately 208,000 patients in age groups of 18 to 90 years. This population denominator was identified and extracted in a prior study focusing on T2D patients and funded by the US Food and Drug Administration (5U01FD005942-05). This population denominator, also known as the raw data cut, was identified by the most inclusive query of the JHMI’s EHR data warehouse, which also included patients with mentions of diabetes in their clinical notes. Each of the identified T2D phenotypes was applied to the overall study population. At total of approximately 164,000 patients were included in at least one of the T2D phenotypes assessed.

Considering the size of the EHR data set, and given the impracticality of reviewing the individual records of almost a quarter of a million patients, no gold standard population was identified for this research. Indeed, the aim of this research study was not to assess the accuracy of the common T2D phenotypes; instead, we aimed to measure the performance of T2D phenotypes given the underlying data quality issues in EHR data repositories.

### T2D Phenotype Definitions

Our assessment included 4 published T2D phenotype definitions, from the CCW, DDC, SUPREME-DM, and eMERGE, as well as 1 unpublished definition (Johns Hopkins University; JHU). Since not all T2D phenotype definitions were defined with the most current *ICD* codes, we converted the DDC, SUPREME-DM, and eMERGE diagnostic phenotypes from *ICD-9* to *ICD-10* [12]. Additionally, not all phenotype definitions using medications had the list of specific RxNorm codes (eg, the DDC and SUPREME-DM phenotypes included only the names of the T2D medication and not the codes). We identified the RxNorm codes for each medication using the National Library of Medicine’s RxNav tool [13]. For the purposes of this study, we selected only those RxNorm codes that were associated with the primary ingredient or ingredients of the medication name in the T2D phenotype definition.

Of the 4 published T2D phenotypes, CCW only included diagnosis codes with a reference period of 2 years in the definition. The DDC, SUPREME-DM, and eMERGE phenotypes included a series of detailed care pathways with diagnosis, medication, and laboratory codes and results within specified time periods. However, the unpublished definition from JHU did not have pathways and was the most inclusive definition, as it included all data types (diagnosis, medication, and laboratory) with wide eligibility criteria to identify patients with T2D.

### Factors Affecting T2D Phenotyping

The data included demographics, T2D-related data types (ie, diagnosis, medication, and laboratory), and the Charlson Comorbidity Index [14]. The demographic data included age, sex (male, female, and other), race (White, Black, Asian, and other), ethnicity (Hispanic/Latino or non-Hispanic/Latino) and patient location (state of Maryland or other locations). The “other” category for sex, race, and ethnicity was primarily composed of missing data entries. Since the JHMI is in Baltimore, Maryland, the majority (169,215/207,813, 81.4%) of our study population was from Maryland and the remainder (38,598/207,813, 18.6%) was from the surrounding states. The outcome measures included the extent of overlap in identifying patients with T2D and the degree of robustness against data quality issues across phenotypes.

### Statistical Analysis

We performed descriptive data analyses across the 5 different phenotypes to identify EHR populations with T2D. We used the *χ*^2^ test for categorical variables and ANOVA for continuous variables. Our analysis included distribution and overlap of the population of interest by diagnosis, medication, and laboratory data types across each of the 5 T2D phenotypes. We introduced methods that simulated or induced data incompleteness, inaccuracy, and lack of timeliness (eg, date shifting) to assess the robustness of each phenotype in capturing the populations of interest. We created unique analytical functions for each data quality issue considering the data types that were applicable across all T2D phenotypes.

To simulate or induce data incompleteness, our procedure randomly dropped codes at 10% increments, from 0% to 100%, for diagnosis, medication, and laboratory codes within each of the T2D phenotypes. Incompleteness was simulated up to 100% as no thresholds of data missingness were known to affect the performance of T2D phenotypes beforehand. Incompleteness was induced for diagnosis, medication, or laboratory codes independently of the other 2 data types. For each increment of incompleteness, T2D phenotypes were reapplied to identify a new (and logically smaller) cohort of patients.

To gauge the impact of inconsistency and inaccuracy in our denominator population for each T2D phenotype, the diagnosis and medication codes were replaced at random at increments of 10% (the same as for incompleteness) from 0% to 100% with another code of the same data type, including T2D and non-T2D codes. We included T2D codes to illustrate expected data quality issues that may impact the phenotypes’ performance. For example, 10% of instances of the *ICD-10* diagnosis code E08 (ie, diabetes mellitus due to underlying condition) were replaced at random with the *ICD-10* code E09 (ie, drug- or chemical-induced diabetes mellitus—endocrine, nutritional, or metabolic disease). For laboratory codes, the laboratory values were induced with a 2% incremental change from −100% to 10% in laboratory results. For example, our procedure for simulated or induced laboratory values yielded results of 5.6% to 5.8% for hemoglobin A_1c_ (LOINC code 55454‐3). Additionally, we simulated or induced inaccuracy in units of laboratory data results. We did this by intentionally converting reported laboratory units from US standards to UK standards; in particular, blood glucose level units in mg/dl were converted to mmol/L, and hemoglobin A_1c_ level from percentage to mmol/mol [15].

For timeliness, we simulated or induced date shifts at increments of 30 to 365 days for all phenotypes. For example, our procedure induced a forward shift on December 1, 2019, by 30 days, shifting the date to December 31, 2019, and so on. Lastly, we also induced compounded data quality issues (incompleteness, inaccuracy, and lack of timeliness) to understand each phenotype’s resistance to changes that occurred across diagnosis, medication, and laboratory codes simultaneously. Induced compounded incompleteness would mean dropping diagnosis, medication, and laboratory codes randomly at increments of 10% up to 100%. Induced compounded inaccuracy would mean replacing diagnosis and medication codes randomly at increments of 10% up to 100% (laboratory codes were not replaced; their values were manipulated instead). Induced compounded lack of timeliness would mean inducing date shifts across diagnosis, medication, and laboratory codes at increments of 30 to 365 days.

SQL queries were written for data extraction. All visualizations and statistical analyses were conducted using R (version 4.2.0; R Foundation for Statistical Computing) [16]. The overall findings are showcased as descriptive data tables, Venn diagrams, and line charts depicting the effect of simulated or induced data quality issues on identifying T2D populations using the common T2D phenotype definitions.

### Ethical Considerations

This study was reviewed and approved by the IRB committee of the Johns Hopkins School of Public Health (00014440). The deidentified population denominator used in this study is from a prior study funded by the US Food and Drug Administration (5U01FD005942-05).

## Results

### Characteristics of the Overall and Phenotype-Identified T2D Populations

Our overall study population included 207,813 patients with T2D from between 2017 and 2019 in the JHMI EHR data. For the purposes of this analysis, we refer to this population as the raw data cut or the overall study population/denominator. The mean age of the overall study population was 62.4 (SD 15.4) years, with 81.4% (n=169,215) of the population residing in the state of Maryland. Women accounted for 51.3% (n=106,704) of the study population, with 31.8% (n=66,073) Black patients and 89.9% (n=186,785) non-Hispanic/Latino patients. The overall population had a mean number of 0.657 (SD 1.61) inpatient visits and 1.01 (SD 3.61) emergency department visits across the study duration. The mean Charlson Comorbidity Index score was 2.17 (SD 2.25). Table 1 shows the overall characteristics of the study population.

**Table 1.** Characteristics of the overall study population (N=207,813).

Characteristics		Values
**Age (years)**		
	Mean (SD)	62.4 (15.4)
	Median (range)	64.0 (18.0-90.0)
**Sex, n (%)**		
	Female	106,704 (51.3)
	Male	101,079 (48.6)
**Race, n (%)**		
Asian	11,644 (5.6)
Black	66,073 (31.8)
	White	109,695 (52.8)
	Other	20,401 (9.8)
**Ethnicity, n (%)**		
	Hispanic/Latino	10,979 (5.3)
	Non-Hispanic/Latino	186,785 (89.9)
	Other	10,049 (4.8)
**Charlson Comorbidity Index score**		
	Mean (SD)	2.17 (2.25)
	Median (range)	1.33 (0-20.0)
**State, n (%)**		
	Maryland	169,215 (81.4)
	Other	38,598 (18.6)
**Inpatient visits (n)**		
	Mean (SD)	0.657 (1.61)
	Median (range)	0 (0-59.0)
**Emergency department visits (n)**		
	Mean (SD)	1.01 (3.61)
	Median (range)	0 (0-415)

**Figure 1.**
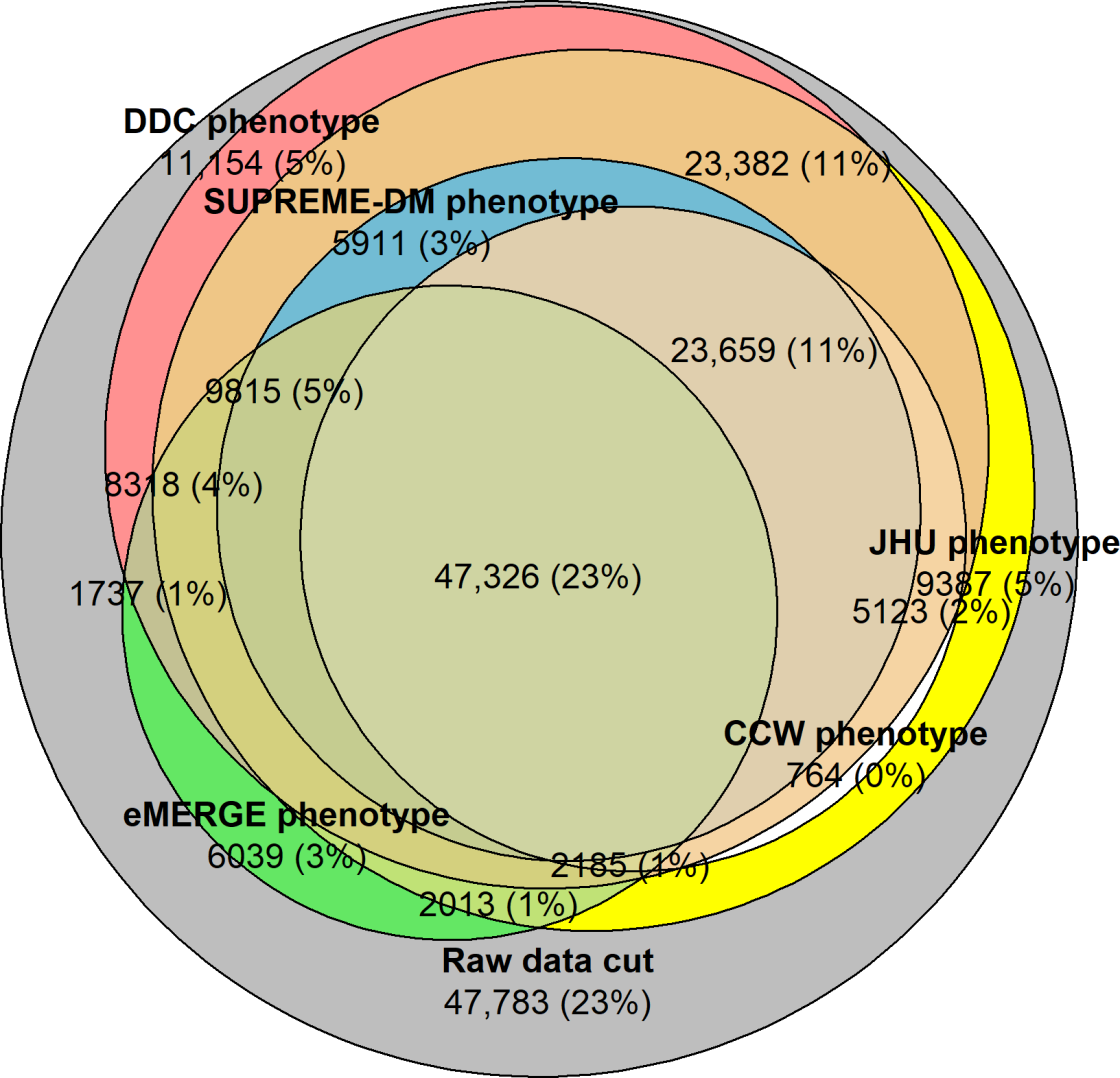
Venn diagram showing overlap of type 2 diabetes populations identified across all phenotype definitions using electronic health record data. CCW: Chronic Conditions Data Warehouse; DDC: Durham Diabetes Coalition; JHU: Johns Hopkins University; SUPREME-DM: Surveillance, Prevention, and Management of Diabetes Mellitus; eMERGE: Electronic Medical Records and Genomics.

T2D phenotypes were applied to the overall study population. The characteristics of the population identified by each phenotype were notably different due to the phenotypes’ varying constraints on medical events and diagnosis, medication, and laboratory codes. The Venn diagram displayed in Figure 1 shows the overlap of the population with T2D across the phenotypes in comparison to the overall study population (ie, the largest, gray circle denotes the raw data cut). These T2D populations were identified using all EHR data types (diagnosis, medication, and laboratory) as needed by the T2D phenotypes. A total of 78% (n=160,030 patients) of the overall study population was identified by at least 1 phenotype, but only 23% (n=47,326) of the overall study population was identified by all T2D phenotypes. DDC identified 139,832 patients with T2D, of which 11,154 (7.98%) were not identified by the other 4 phenotype definitions. Of the 89,772 patients with T2D identified by SUPREME-DM, there were 5911 (3%) patients with T2D that were also identified by the DDC and JHU phenotypes. Additionally, 23,659 (12%) patients were identified by all phenotypes except eMERGE. Additional details of population overlap counts are available in Multimedia Appendix 1, Table S1.

The distributions of T2D populations identified across phenotype definitions by diagnosis, medication, and laboratory data types were separately calculated (Table 2). The SUPREME-DM phenotype identified the most patients (n=33,268) across all 3 data types, followed by eMERGE with 30,573 patients. Zero patients were observed for some phenotypes when using specific data types. The zero observations were due to the representation of the data types as defined by the criteria of each phenotype definition. For example, the eMERGE phenotype does not have a pathway to identify a patient with T2D based on diagnosis code only, medication code only, or medication and laboratory codes only. Hence, we observed zero patients with diagnosis only, medication only, or medication and laboratory only for eMERGE (Table 2). In the case of CCW, since the phenotype does not include medication or laboratory codes in its definition, zero patients were observed when medication or laboratory codes were required for the identification of T2D patients (Table 2).

We measured the effect of simulated or induced data quality issues of completeness, accuracy, and timeliness for diagnosis, medication, and laboratory data across all T2D phenotypes. The following results include the percentage of patients identified by each phenotype while simulating data quality issues using diagnosis codes. Figures S1 to S3 in Multimedia Appendix 1 show the same diagnosis results but depict the frequency of patients identified by each phenotype. Figures S4 to S17 in Multimedia Appendix 1 show results as the percentages and frequencies of patients identified by each phenotype while simulating data quality issues using medication and laboratory data types.

**Table 2.** Distribution of type 2 diabetes (T2D) populations identified by T2D phenotype definitions using different combinations of data types.

Data type	DDC^a^ (n=139,832), n	SUPREME-DM^b^ (n=89,772), n	eMERGE^c^ (n=77,977), n	JHU^d^ (n=139,231), n	CCW^e^ (n=79,967), n
Diagnosis	40,133	15,983	0	75,027	79,967
Medication	15,511	0	0	5874	0
Laboratory	5973	1907	10,467	2711	0
Diagnosis and medication	26,993	24,328	31,114	21,336	0
Diagnosis and laboratory	21,546	13,237	5823	19,452	0
Medication and laboratory	987	1049	0	83	0
Diagnosis, medication, and laboratory	28,687	33,268	30,573	14,748	0

a DDC: Durham Diabetes Coalition.

b SUPREME-DM: Surveillance, Prevention, and Management of Diabetes Mellitus.

c eMERGE: Electronic Medical Records and Genomics.

d JHU: Johns Hopkins University.

e CCW: Chronic Conditions Data Warehouse.

### Data Quality Issues

#### Completeness

**Figure 2.**
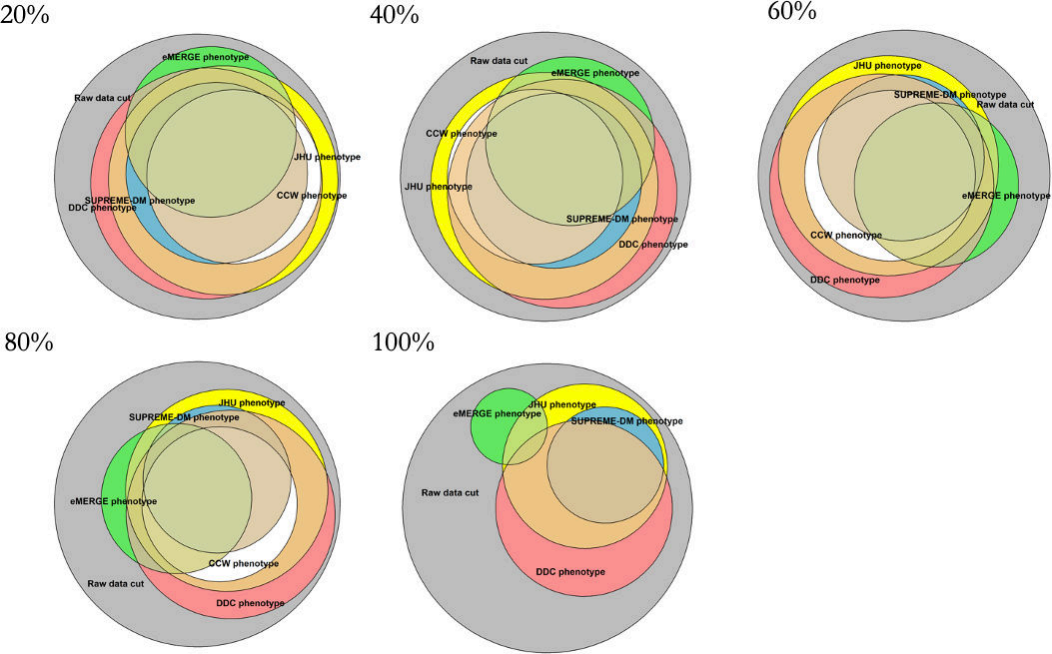
Overall population identified by each of the type 2 diabetes phenotype definitions when diagnosis codes were dropped from 20% to 100% to simulate increasing incompleteness of diagnosis codes. CCW: Chronic Conditions Data Warehouse; DDC: Durham Diabetes Coalition; eMERGE: Electronic Medical Records and Genomics; JHU: Johns Hopkins University; SUPREME-DM: Surveillance, Prevention, and Management of Diabetes Mellitus.

To depict the impact of the simulated or induced incompleteness of diagnosis codes on the identified population using the T2D phenotypes, Figure 1 was recreated at increasing levels of data incompleteness (Figure 2). As the percentage of induced missing diagnosis codes increased, the size of the T2D population identified by each phenotype decreased. The CCW phenotype saw the largest decline in the identified population with increasing incompleteness. At 100% induced incompleteness, there were no T2D patients identified by CCW, as the CCW phenotype relies entirely on diagnosis codes for the identification of T2D patients. Lastly, the eMERGE, SUPREME-DM, DDC, and JHU phenotypes continued to identify patients with T2D despite 100% incompleteness of diagnosis codes (Figure 2).

**Figure 3.**
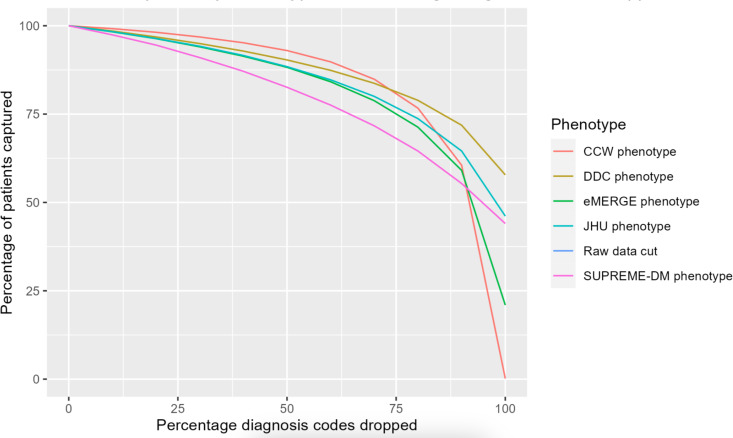
Percent of type 2 diabetes population identified by each type 2 diabetes phenotype definition with increasing incompleteness of diagnosis codes. CCW: Chronic Conditions Data Warehouse; DDC: Durham Diabetes Coalition; eMERGE:lectronic Medical Records and Genomics; JHU: Johns Hopkins University; SUPREME-DM: Surveillance, Prevention, and Management of Diabetes Mellitus.

Figure 3 shows the decrease in the percentage of T2D population identified by each phenotype when diagnostic incompleteness was induced from 0% to 100%, that is, diagnosis codes from the *ICD-10* were dropped in increments of 10%. All phenotype definitions showed a similar decrease in the percentage of patients from 0% to approximately 80% of diagnostic incompleteness. The eMERGE and CCW phenotypes saw significant declines in T2D population sizes when the induced incompleteness increased from 80% to 100%. At 100% incompleteness, CCW identified no patients and eMERGE identified only 21% (16,348/77,977) of the patients with T2D, consistent with Table 2 and Figure 2.

#### Accuracy

**Figure 4.**
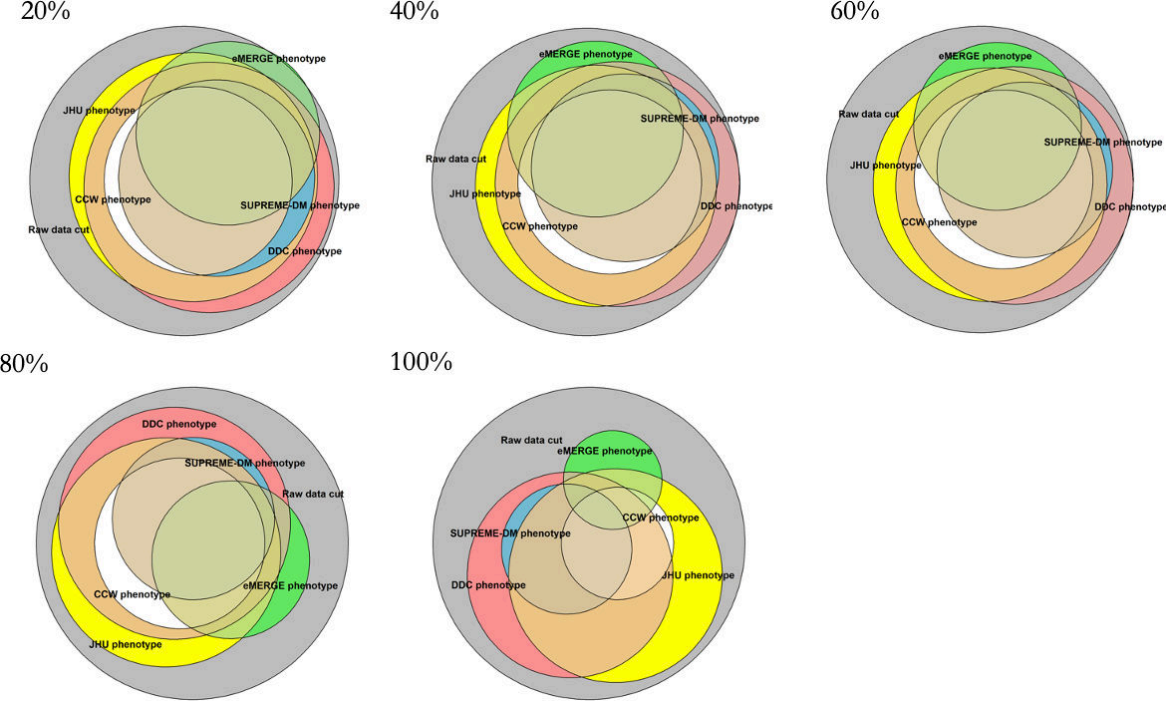
Overall population identified by each of the type 2 diabetes phenotype definitions when diagnosis codes are replaced at random to simulate increasing diagnostic inaccuracy. CCW: Chronic Conditions Data Warehouse; DDC: Durham Diabetes Coalition; eMERGE: Electronic Medical Records and Genomics; JHU: Johns Hopkins University; SUPREME-DM: Surveillance, Prevention, and Management of Diabetes Mellitus.

Figures4  5 show the overlap and percentages, respectively, of T2D populations identified by each phenotype definition with an increasing percentage of induced replacement of diagnosis codes at random. All phenotype definitions were impacted and continued to identify populations with T2D even with 100% induced inaccuracy despite reductions overall. The CCW, SUPREME-DM, and eMERGE phenotypes showed the greatest decrease when 75%‐100% of the diagnosis codes were replaced, resulting in identification of only 27%‐45% of the patients with T2D.

**Figure 5.**
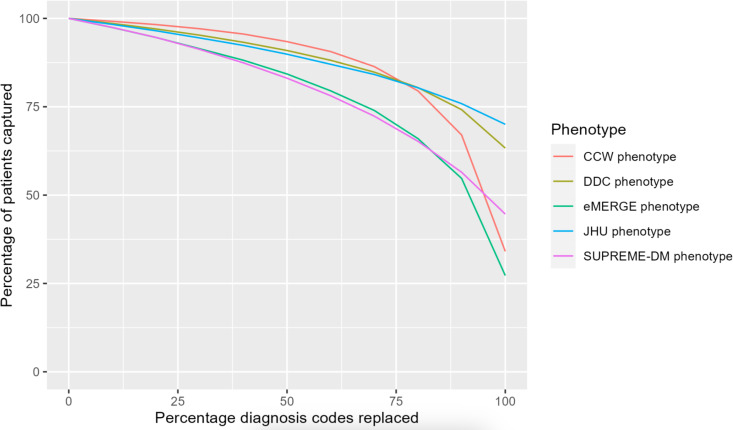
Percentage of type 2 diabetes population identified by each type 2 diabetes phenotype definition with increasing inaccuracy of diagnosis codes. CCW: Chronic Conditions Data Warehouse; DDC: Durham Diabetes Coalition; eMERGE: Electronic Medical Records and Genomics; JHU: Johns Hopkins University; SUPREME-DM: Surveillance, Prevention, and Management of Diabetes Mellitus.

#### Timeliness

**Figure 6.**
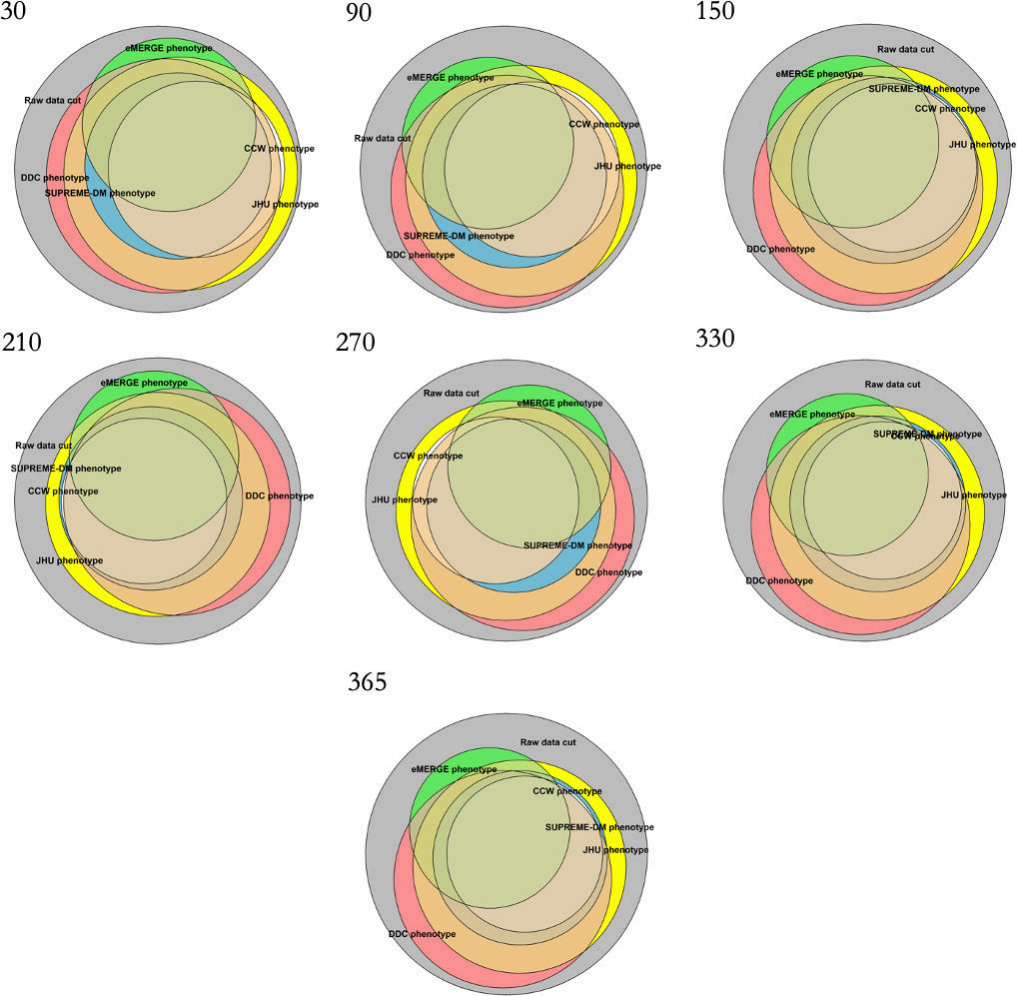
Overall population identified by each of the type 2 diabetes phenotype definitions with shifts in timeliness of diagnostic data ranging from 30 to 365 days. CCW: Chronic Conditions Data Warehouse; DDC: Durham Diabetes Coalition; eMERGE: Electronic Medical Records and Genomics; JHU: Johns Hopkins University; SUPREME-DM: Surveillance, Prevention, and Management of Diabetes Mellitus.

**Figure 7.**
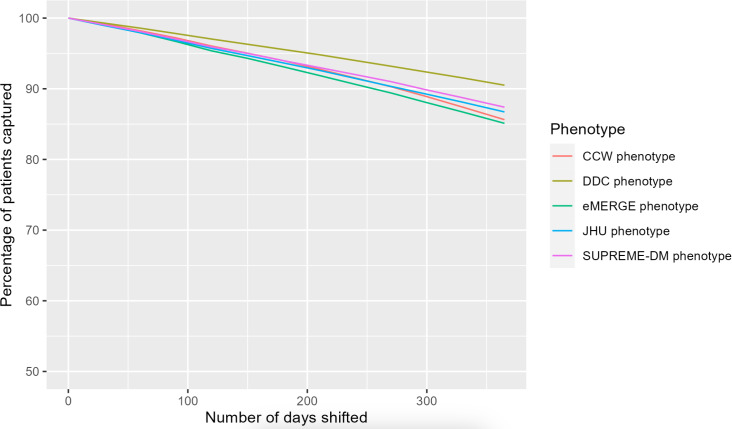
Percentage of type 2 diabetes population identified by each type 2 diabetes phenotype definition with an increasing shift in diagnostic timeliness (ie, number of days shifted). CCW: Chronic Conditions Data Warehouse; DDC: Durham Diabetes Coalition; eMERGE: Electronic Medical Records and Genomics; JHU: Johns Hopkins University; SUPREME-DM: Surveillance, Prevention, and Management of Diabetes Mellitus.

All T2D phenotypes were similarly impacted by simulated or induced shifts in the date of recorded diagnosis from 30 days to 365 days. The patterns of overlap in population were similar across all phenotypes with each incremental date shift (Figure 6). The percentage of patients identified with T2D showed a decrease over time, however, with at least 85% (176,641/207,813) of the patients with T2D identified during the progression of a year across all phenotypes. The data quality issue of timeliness showed the least impact in the DDC phenotype (Figure 7).

#### Compounded Data Quality Issue: Completeness

**Figure 8.**
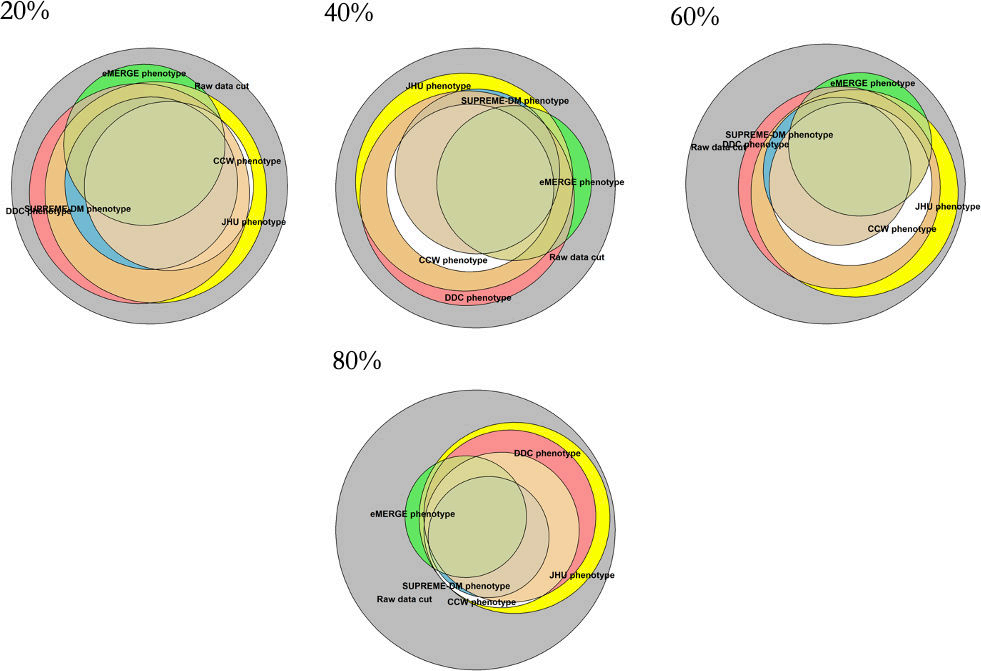
Overall population identified by each of the type 2 diabetes phenotype definitions with compounded increasing incompleteness (diagnostic, medication, and laboratory codes). CCW: Chronic Conditions Data Warehouse; DDC: Durham Diabetes Coalition; eMERGE: Electronic Medical Records and Genomics; JHU: Johns Hopkins University; SUPREME-DM: Surveillance, Prevention, and Management of Diabetes Mellitus.

Figures8  9 show the overlap and percentages, respectively, of T2D populations identified by each phenotype definition with an increasing percentage of induced compounded incompleteness across diagnosis, medication, and laboratory codes. All phenotype definitions were impacted and continued to identify populations with T2D until 100% induced incompleteness, as expected. While all phenotypes exhibited similar rates of decrease with increased compounded incompleteness, CCW was the most robust to incompleteness. SUPREME-DM was the least resistant to induced compounded incompleteness. Figures S18 and S21 in Multimedia Appendix 1 provide results for the percentages and frequencies of patients identified by each phenotype while simulating the compounded data quality issues of inaccuracy and lack of timeliness.

**Figure 9.**
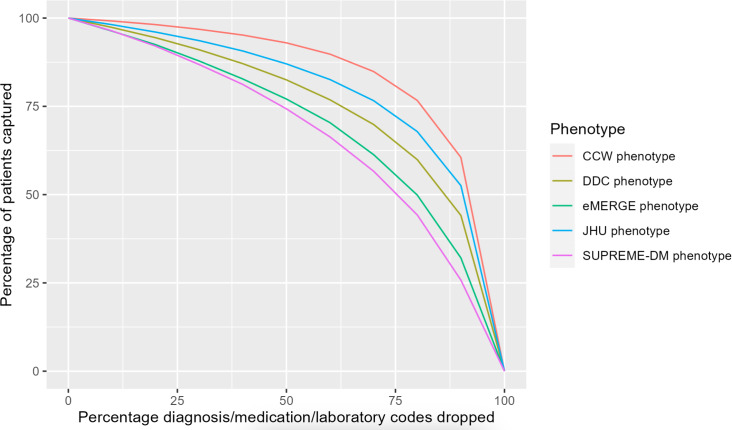
Percentage of type 2 diabetes population identified by each type 2 diabetes phenotype definition with compounded increasing incompleteness (diagnostic, medication, and laboratory codes). CCW: Chronic Conditions Data Warehouse; DDC: Durham Diabetes Coalition; eMERGE: Electronic Medical Records and Genomics; JHU: Johns Hopkins University; SUPREME-DM: Surveillance, Prevention, and Management of Diabetes Mellitus.

## Discussion

T2D is a common chronic disease with no known universally defined phenotype definition. This is further complicated by lack of assessments that are necessary to understand the quality of data in real-world health care settings against common phenotypes. In our study, we investigated how data quality issues of completeness, accuracy, and timeliness may impact the implementation of existing T2D phenotypes using different data types of diagnosis, medication, and laboratory codes extracted from common clinical data sources such as EHRs. Each data quality issue and phenotype definition may present unique implications at an unpredictable scale in any given health care setting. Collectively, these variables can pose challenges in identifying an eligible denominator population and implementing study population enrollment strategies for epidemiological studies, population health and management, disease surveillance, financial operations, and policies for populations with T2D.

Although each phenotype has distinct characteristics, our results showed considerable overlap in the population identified with T2D across the phenotype definitions (Figure 1 and Table 2). Given the uniqueness of the CCW phenotype, which only includes diagnosis codes, the CCW phenotype identified approximately 38.4% (79,967/207,813) of the overall study population with T2D, whereas the DDC phenotype, which was inclusive of all data types, identified approximately 67.2% (139,832/207,813) of the total population with T2D. This was similar to the 66.9% (139,231/207,813) of the total population identified by the Hopkins phenotype. Additionally, we observed that the T2D phenotype definitions of SUPREME-DM and eMERGE had similar characteristics and therefore resulted in the identification of approximately 43.2% (89,772/207,813) and 37.5% (77,977/207,813) of the overall study population, respectively. Moreover, when analyzing the distribution of population by data types, the identified populations were significantly different, with no noticeable patterns.

This study revealed significant findings resulting from simulated or induced data quality issues on the overall study population using EHR data across different T2D phenotype definitions. Induced incompleteness of diagnostic data showed the least impact on the DDC phenotype, however, identifying only approximately 60% of the population with T2D at 100% diagnostic incompleteness. The CCW phenotype was the most impacted, as 100% of the diagnosis codes were missing with 100% induced incompleteness given the characteristics of its phenotype definition. With more incomplete data, the uniqueness of the population identified by each phenotype may also shift, thereby showing a decrease in overlap of the population with T2D across all phenotypes. Although trends displayed by phenotype definitions of Hopkins and SUPREME-DM may seem similar, the quantification of the slightest differences in trends can have significant population health implications, resulting in compromised financial and logistical (eg, staffing needs) outcomes. Thus, it is important to understand the resistance of data quality issues across phenotypes given different data types.

The results for induced inaccuracy were similar to those for completeness, but data quality issues for timeliness showed a different trend. The phenotypes of eMERGE, CCW, and SUPREME-DM showed significant decreases in the number of identified patients when the majority of the diagnosis codes were replaced. However, induced timeliness continued to capture significant numbers of patients for all phenotypes, at well above 85%. While the impact of induced inaccuracies of laboratory values (in the negative direction) was mitigated by other pathways of the phenotypes, overall, completeness and accuracy showed a much larger impact on the identification of the population with T2D. The trends for the data quality issues of completeness and accuracy paint a similar picture, which may translate into considerations for population health interventions. The effect of data quality on identifying T2D populations using commonly available T2D phenotypes can affect a variety of interventions and outcomes, such as clinical research, financial analysis, staffing needs, and logistical issues, to name a few.

Lastly, induced compounded (diagnosis, medication, and laboratory) data quality issues showed different trends compared to induced single component (diagnosis, medication, or laboratory) data quality issues. Overall, all phenotypes captured fewer patients when there were compounded data quality issues compared to single-component data quality issues. The CCW phenotype was the most resistant phenotype across compounded incompleteness, inaccuracy, and lack of timeliness. The CCW phenotype’s robustness to compounded incompleteness owes to its reliance solely on diagnosis codes; other phenotypes with medication and laboratory components faced steeper drops in patients captured when medication and laboratory codes were both incomplete. When it came to induced compounded inaccuracy, the CCW phenotype demonstrated the greatest resistance until 80% of both diagnosis and medication codes were replaced, at which point the Hopkins phenotype became the most robust (Figure S19 in Multimedia Appendix 1). Similarly, compounded lack of timeliness became a bigger problem for phenotypes that rely on time interval for not only diagnosis, but also medication and laboratory codes. Simulating compounded data quality issues has implications for evaluating the fit of phenotype definitions for various data sources and availabilities of multiple data elements. For instance, if a particular data source only has reliable diagnosis codes, then the CCW phenotype would be the most robust. However, it may come with more false positives due to its sole reliance on diagnosis codes.

Our study highlights the importance of understanding the effects of data quality issues on phenotypes, particularly for common diseases such as T2D. The results from our study are novel and can inform how to better identify denominators for a given purpose, which may be beneficial for both research and operational decisions. Although there is no gold standard data set to compare and analyze baseline thresholds of impact on phenotypes, the results of this study can be used by any researcher using real-world data. Additionally, our study describes methods for assessment of multiple data quality issues that can be applied simultaneously in any clinical and health care research setting.

Our study has some limitations. First, the results discussed are based on the EHR data of JHMI over a period of 3 years; hence, generalizability of the findings may be limited to populations like that of this study population. That said, it may also be not applicable to populations of much smaller sample sizes. Second, our study results should be tested against the epidemiological trends and spread of T2D over time. Third, our study did not measure the embedded issues of data quality problems (ie, only simulated or induced data quality issues); understanding and resolving these issues at the beginning of any study can be vital. Fourth, we did not study other data quality dimensions such as concordance and provenance, the assessment of which can be important. Fifth, all T2D phenotypes used in this study relied on diagnosis, medication, and laboratory codes to identify T2D patients; however, some patients may only have clinically diagnosed information in physician notes (ie, unstructured data or free text) that will be missed using structured diagnosis, medication, or laboratory codes. As a result, there may be some false negatives that may have been excluded from our overall study population despite incorporating the most inclusive criteria for the raw data cut. Sixth, we explored compounded data incompleteness, inaccuracy, and timeliness by dropping multiple data types together at the same increments for each data quality domain. However, there could be alternative versions of compounded simulation where diagnosis, medication, and laboratory data qualities are induced at different levels simultaneously; this may be of interest for future research. Seventh, we assessed the robustness of the phenotypes as they were designated. However, future research could stratify the analysis by the severity of the condition of interest, as severity could impact diagnosis, medication, and laboratory coding behavior and quality. And lastly, our study did not consider any existing data quality thresholds to assess epidemiological impacts on T2D patients using EHRs (eg, comparing national rates of T2D in a neighborhood vs T2D rates identified using EHR data of patients residing in the same neighborhood).

As a result of these observations, we believe that there is a growing need in the United States for a standardized phenotype definition to identify T2D populations while considering the challenges of data quality issues in real-world data, such as from EHRs. The universal phenotype definition should have the ability to integrate features of EHR data while being resistant to common data quality issues. Such a phenotype definition ought to also consider factors that may introduce racial bias and disparities, which eventually may result in health inequities. And lastly, this fundamental definition must also consider integration and interoperability with other data sources, such as claims data, and alignment with existing data interoperability standards. Our research hopes to inspire T2D subject matter experts, at least, to begin conversations toward creating a universal definition for a disease that is extremely common. That said, there is opportunity for additional research that ties issues of data quality with those of phenotypes, data types, and data sources.

Our research provides novel results to understand the effect of data quality issues in data sources like EHRs to identify T2D population-level groups of interest using commonly available T2D phenotype definitions. The study results can inform research or operational efforts using large clinical data repositories to identify T2D populations. Lastly, the study findings can inform efforts to consolidate T2D phenotypes in the near future.

## Supplementary material

10.2196/56734 Multimedia Appendix 1 Table S1 and Figures S1-S21.

## Abbreviations

CCW: Chronic Conditions Data Warehouse
DDC: Durham Diabetes Coalition
EHR: electronic health record
eMERGE: Electronic Medical Records and Genomics
*ICD*: *International Classification of Diseases*
IRB: institutional review board
JHMI: Johns Hopkins Medical Institution
JHU: Johns Hopkins University
LOINC: Logical Observation Identifiers Names and Codes
SUPREME-DM: Surveillance, Prevention, and Management of Diabetes Mellitus
T2D: type 2 diabetes
