# Supplementary material for: Assessing the Effect of Electronic Health Record Data Quality on Identifying Patients With Type 2 Diabetes: Cross-Sectional Study
Source: JMIR Med Inform. 2024 Aug 27;12:e56734. doi: 10.2196/56734 (PMC11370182; doi:10.2196/56734)
Supplement: Multimedia Appendix 1 [file medinform-v12-e56734-s001.docx]

Assessing the Effect of Electronic Health Record Data Quality on
Identifying Patients with Type 2 Diabetes

**Priyanka D. Sood, DrPH, MPH, B.S., ^1^, Star Liu, M.S.^2^, Harold Lehmann, M.D., Ph.D.^2^, Hadi Kharrazi, M.D., Ph.D. ^1,2^**

**^1^Johns Hopkins Bloomberg School of Public Health, Baltimore, MD;
^2^Johns Hopkins School of Medicine, Baltimore, MD**

# Appendix

**Appendix Table 1.** Distribution and overlap of T2D population across phenotype definitions.

| **Phenotype Overlap** | **N*** |
| --- | --- |
| Raw Data Cut | 47,783 |
| Raw Data Cut & DDC | 11,154 |
| Raw Data Cut & SDM | 643 |
| Raw Data Cut & eMERGE | 6,039 |
| Raw Data Cut & Hopkins | 9,387 |
| Raw Data Cut & CCW & Hopkins | 764 |
| Raw Data Cut & DDC & SDM | 1,218 |
| Raw Data Cut & DDC & eMERGE | 1,737 |
| Raw Data Cut & DDC & Hopkins | 23,382 |
| Raw Data Cut & SDM & eMERGE | 4 |
| Raw Data Cut & SDM &Hopkins | 338 |
| Raw Data Cut & eMERGE &Hopkins | 2,013 |
| Raw Data Cut & CCW & DDC & SDM | 1 |
| Raw Data Cut & CCW & DDC & Hopkins | 5,123 |
| Raw Data Cut & CCW & SDM & Hopkins | 473 |
| Raw Data Cut & CCW & eMERGE & Hopkins | 156 |
| Raw Data Cut & DDC & SDM & eMERGE | 3 |
| Raw Data Cut & DDC & SDM & Hopkins | 5,911 |
| Raw Data Cut & DDC & eMERGE & Hopkins | 8,318 |
| Raw Data Cut & SDM & eMERGE & Hopkins | 101 |
| Raw Data Cut & CCW & DDC & SDM & Hopkins | 23,659 |
| Raw Data Cut & CCW & DDC & eMERGE & Hopkins | 2,185 |
| Raw Data Cut & CCW & SDM & eMERGE & Hopkins | 280 |
| Raw Data Cut & DDC & SDM & eMERGE & Hopkins | 9,815 |
| Raw Data Cut & CCW & DDC & SDM & eMERGE & Hopkins | 47,326 |

* Any overlap measures that resulted in zero patient count were removed from this table.


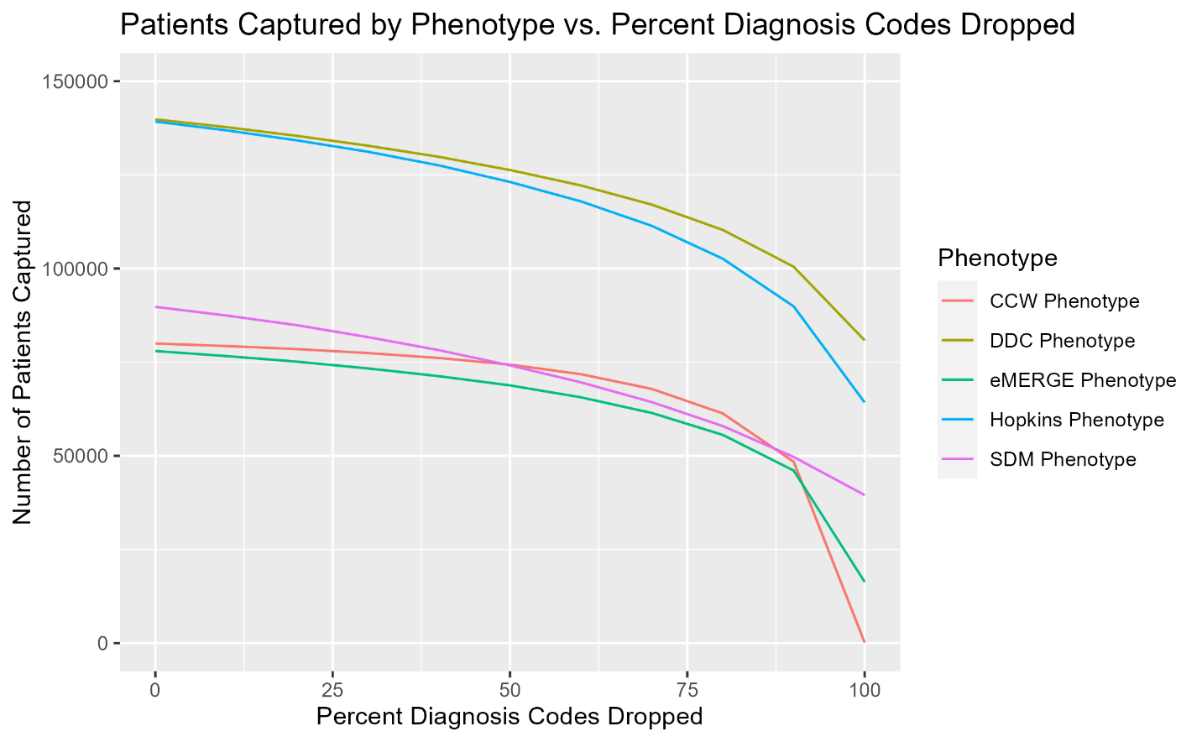


**Appendix Figure 1**. Number of T2D population identified by each T2D phenotype
definition with increasing incompleteness of diagnosis codes.


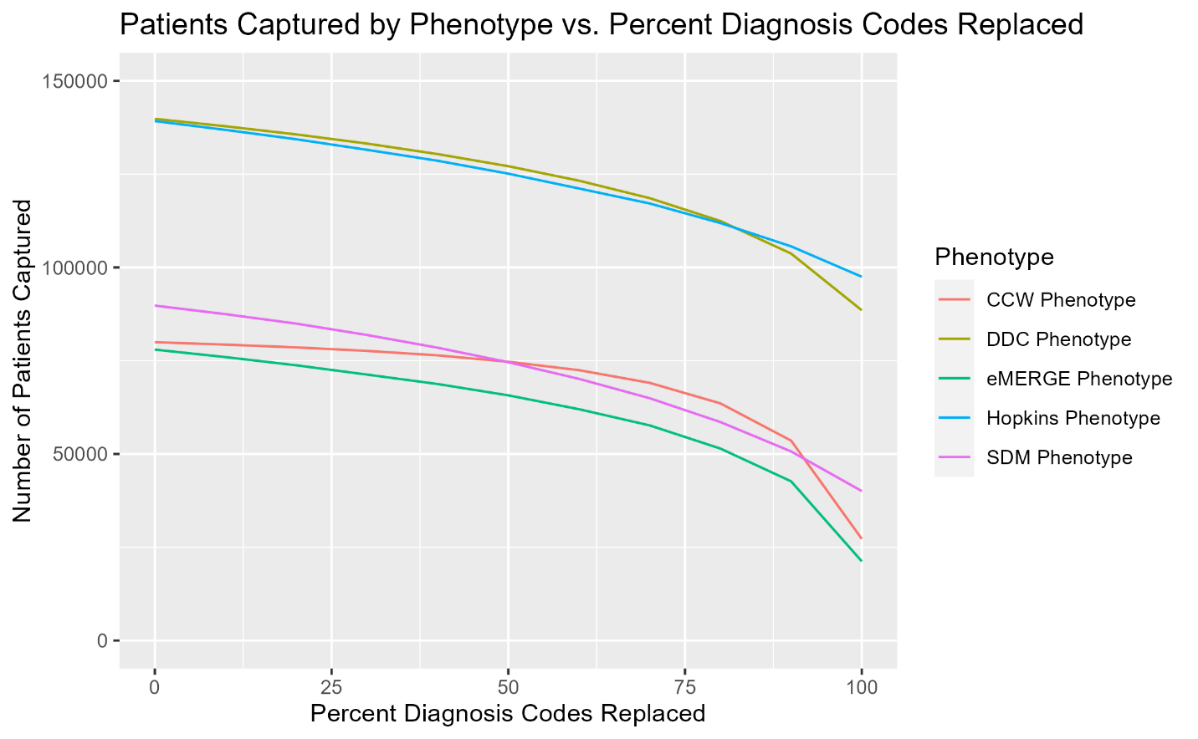


**Appendix Figure 2.** Number of T2D population identified by each T2D phenotype definition with
increasing inaccuracy of diagnosis codes.


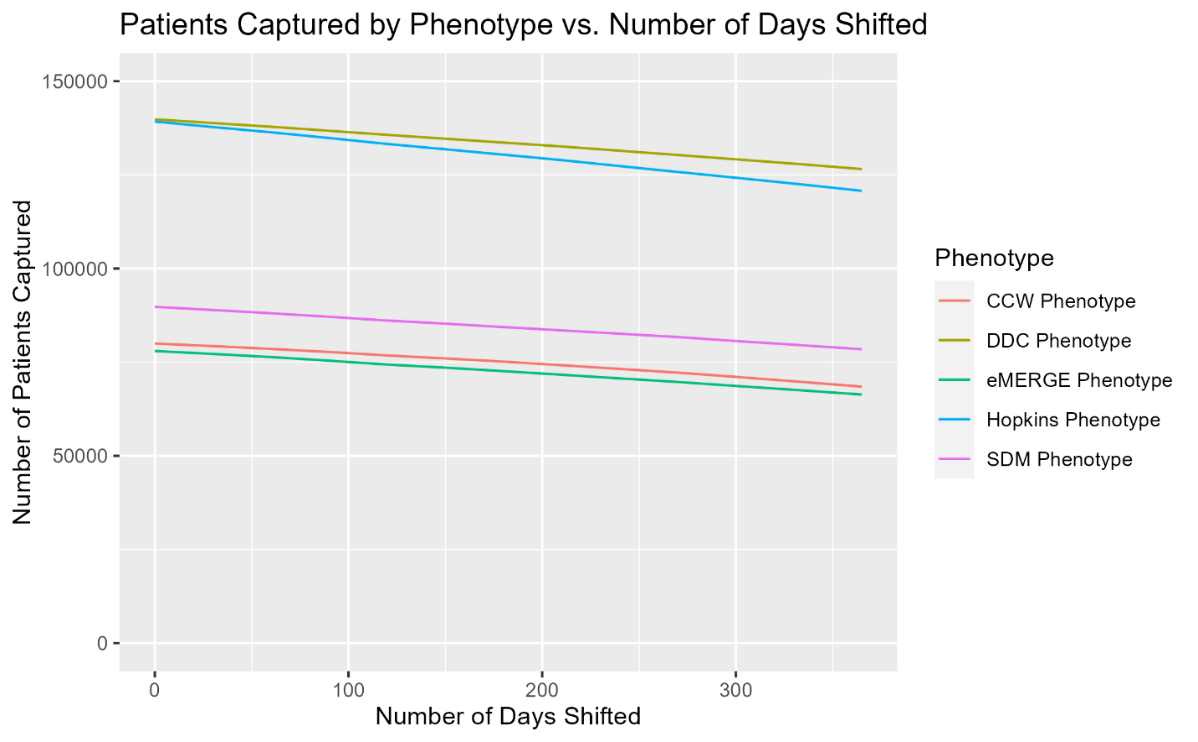


**Appendix Figure 3.** Number of T2D population identified by each T2D phenotype definition
with increase shift in diagnosis timeliness (i.e., days shift).


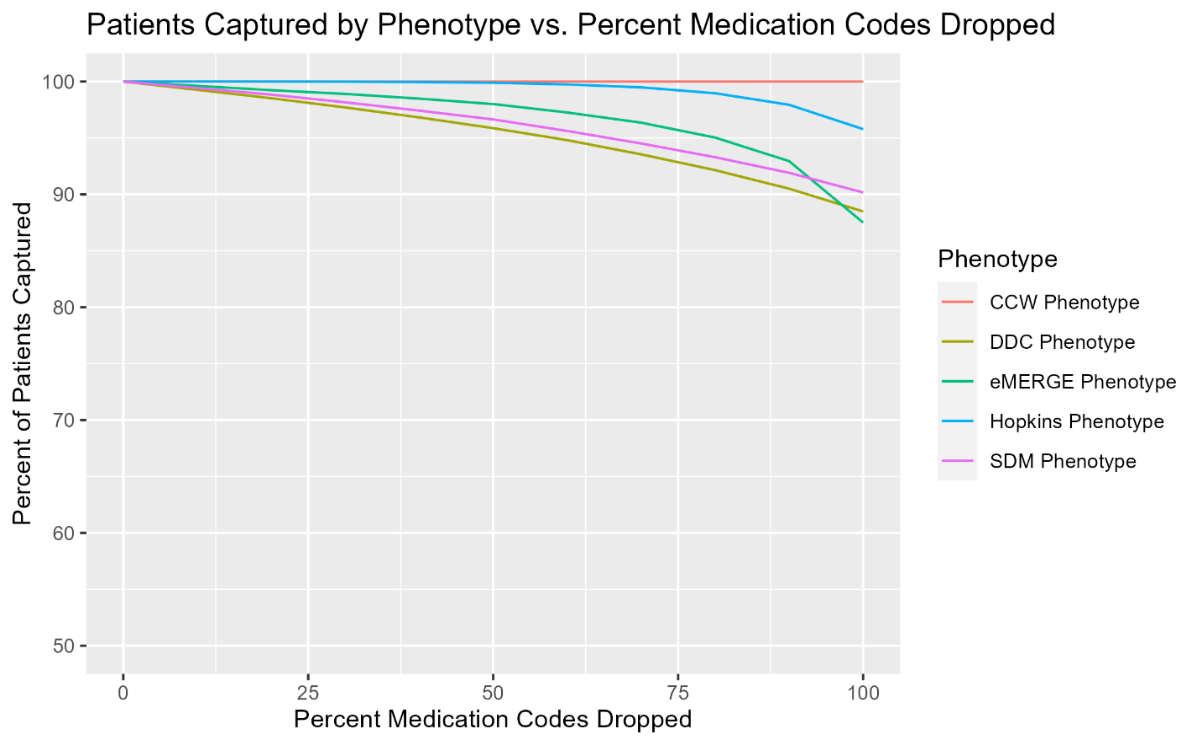


**Appendix Figure 4**. Percent of T2D population identified by each T2D phenotype
definition with increasing incompleteness of medication codes.


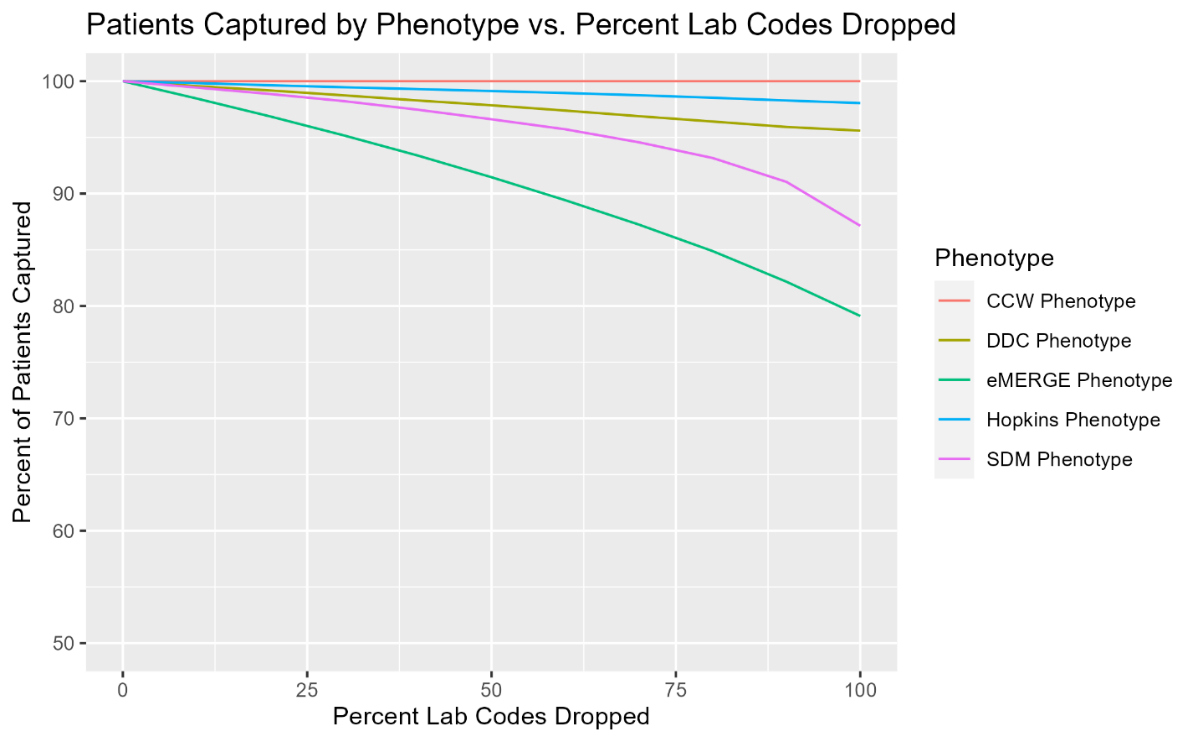


**Appendix Figure 5**. Percent of T2D population identified by each T2D phenotype
definition with increasing incompleteness of laboratory results.


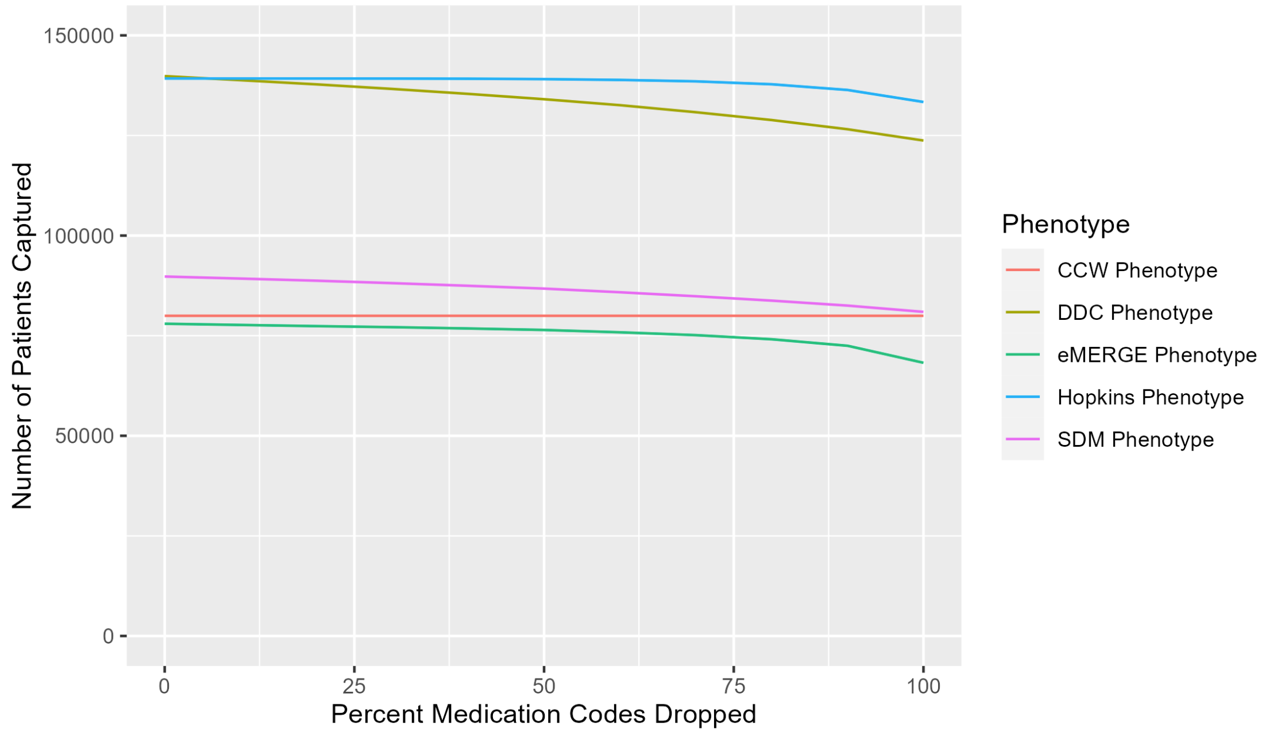


**Appendix Figure 6**. Number of T2D population identified by each T2D phenotype
definition with increasing incompleteness of medication codes.


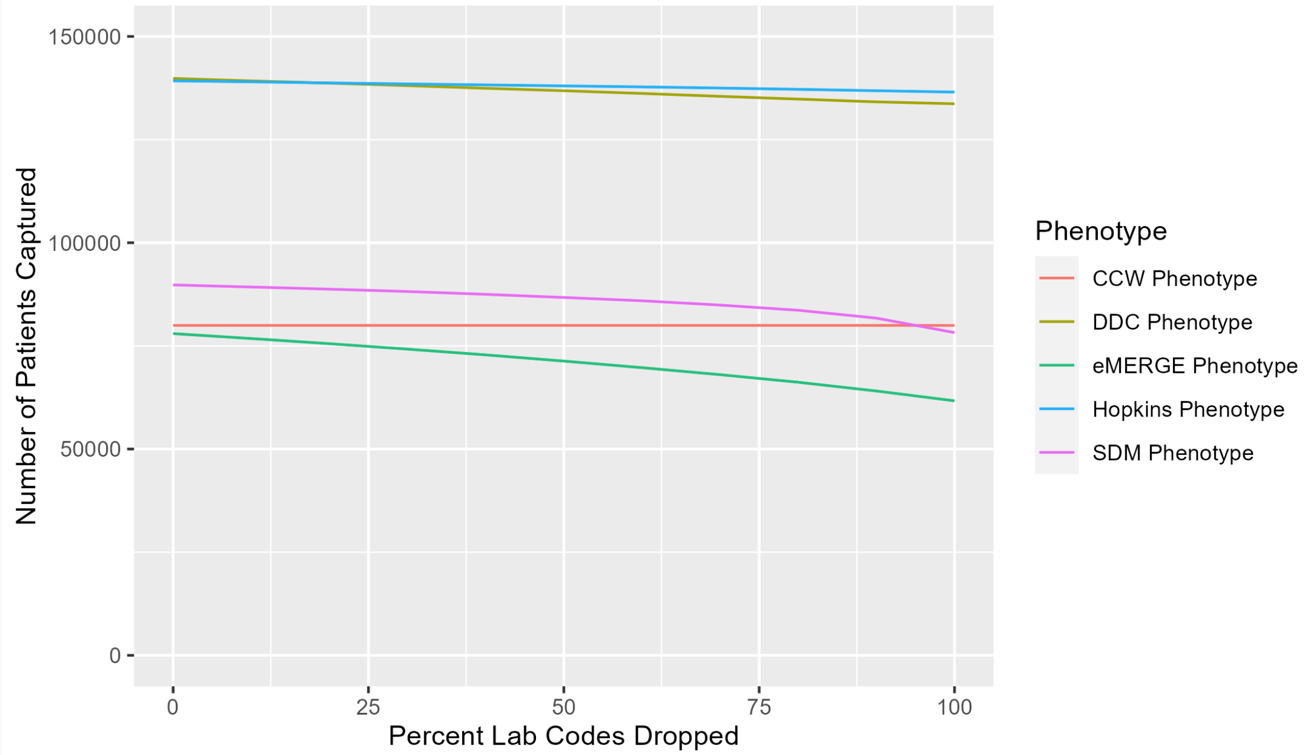


**Appendix Figure 7**. Number of T2D population identified by each T2D phenotype
definition with increasing incompleteness of laboratory codes.


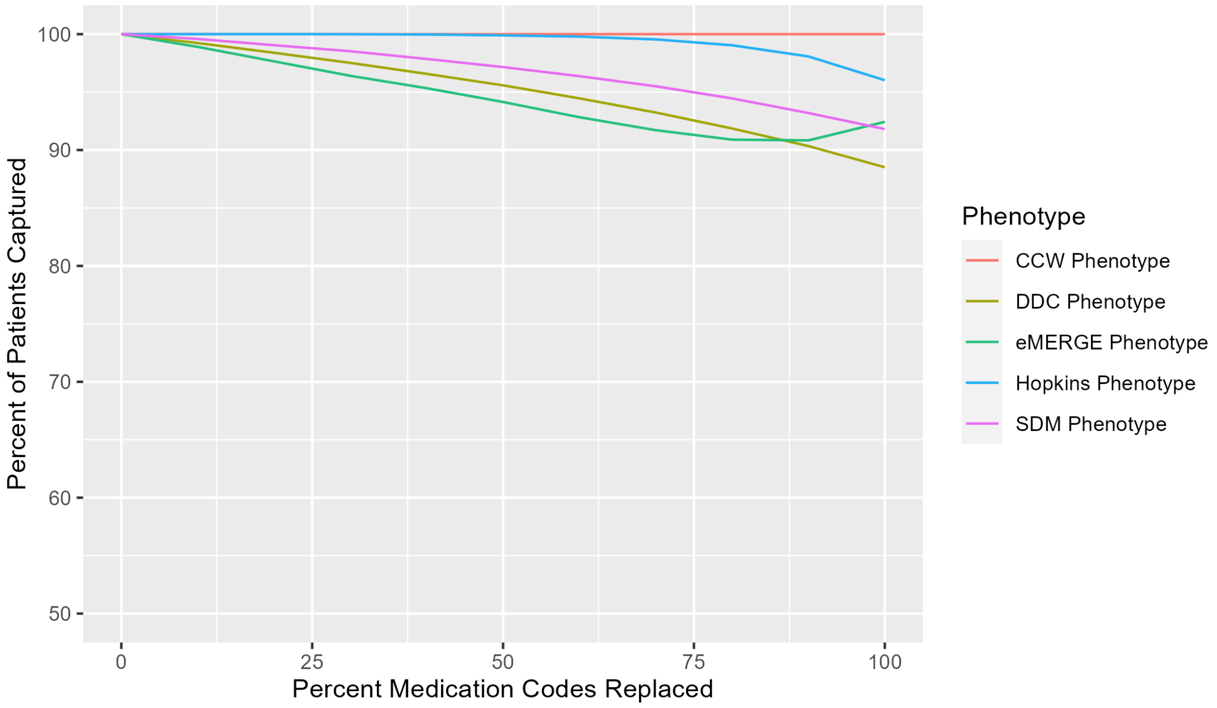


**Appendix Figure 8.** Percent of T2D population identified by each T2D phenotype definition with
increasing inaccuracy of medication codes.


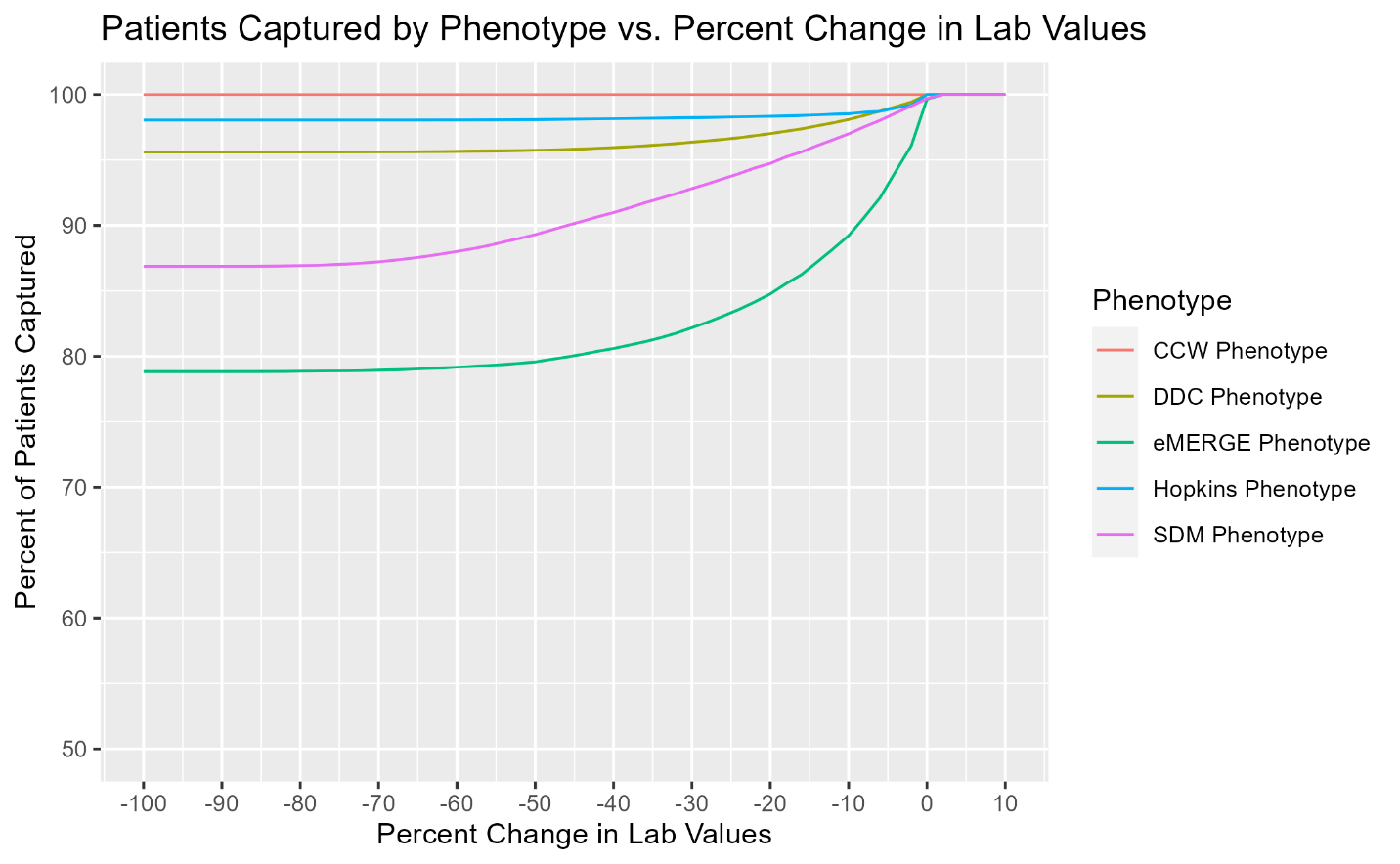


**Appendix Figure 9.** Percent of T2D population identified by each T2D phenotype definition with
increasing inaccuracy of laboratory values.


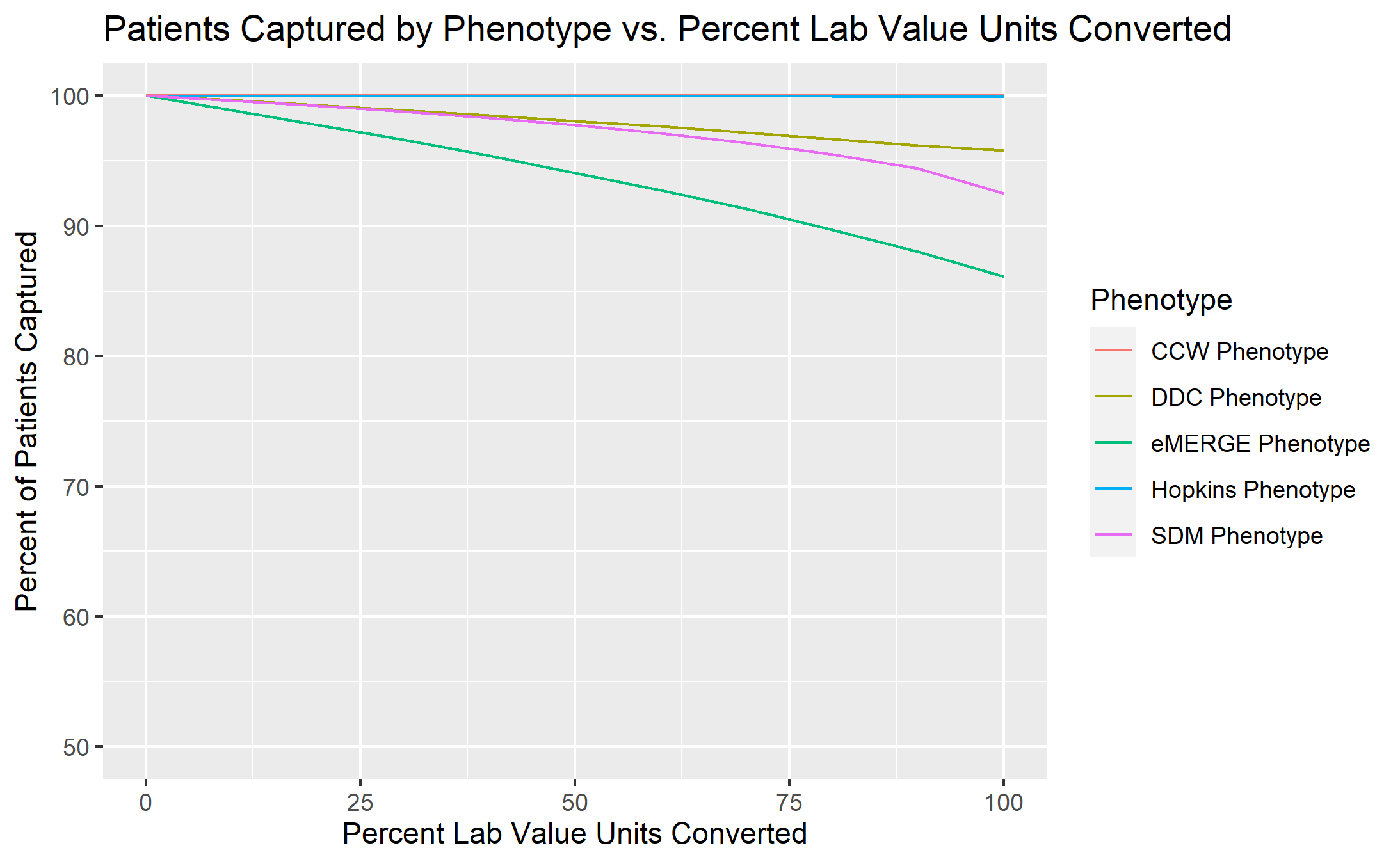


**Appendix Figure 10.** Percent of T2D population identified by each T2D phenotype definition with
increasing inaccuracy of units of laboratory results.


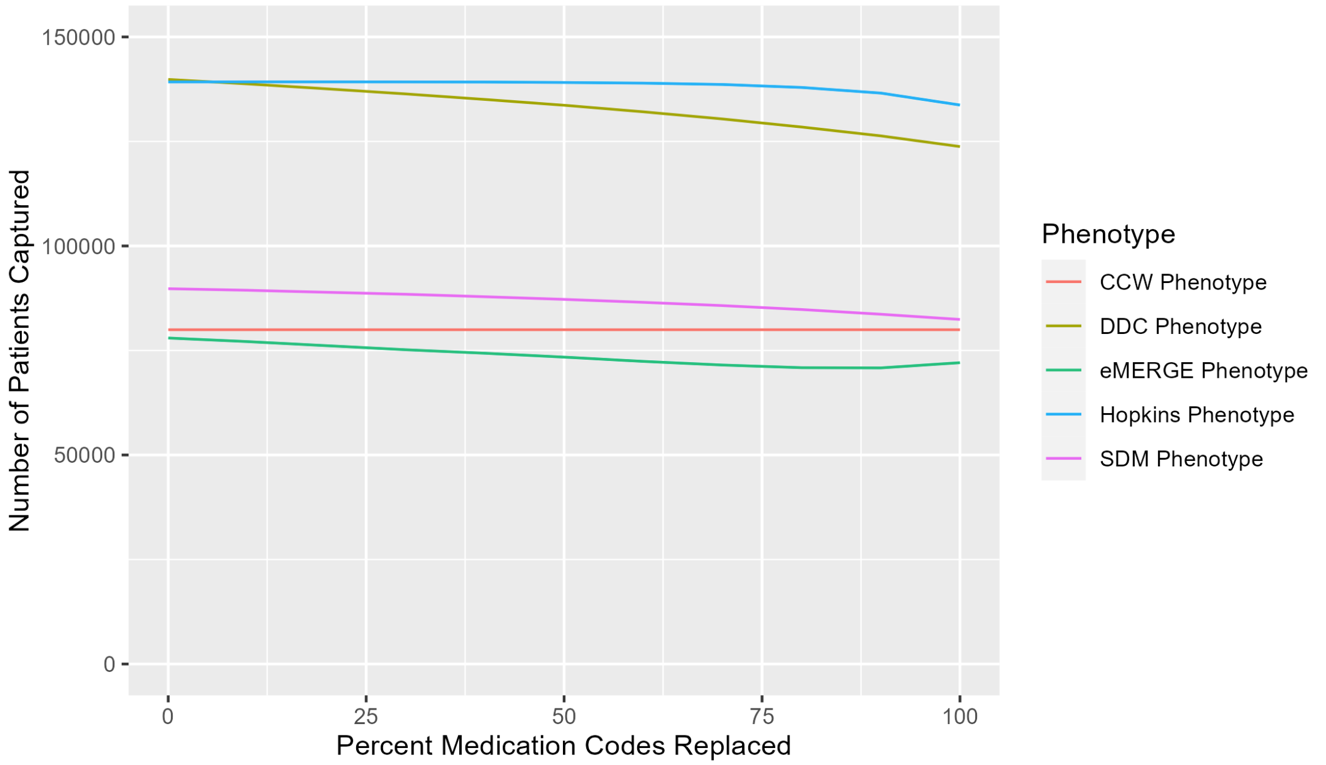


**Appendix Figure 11**. Number of T2D population identified by each T2D phenotype
definition with increasing inaccuracy of medication codes.


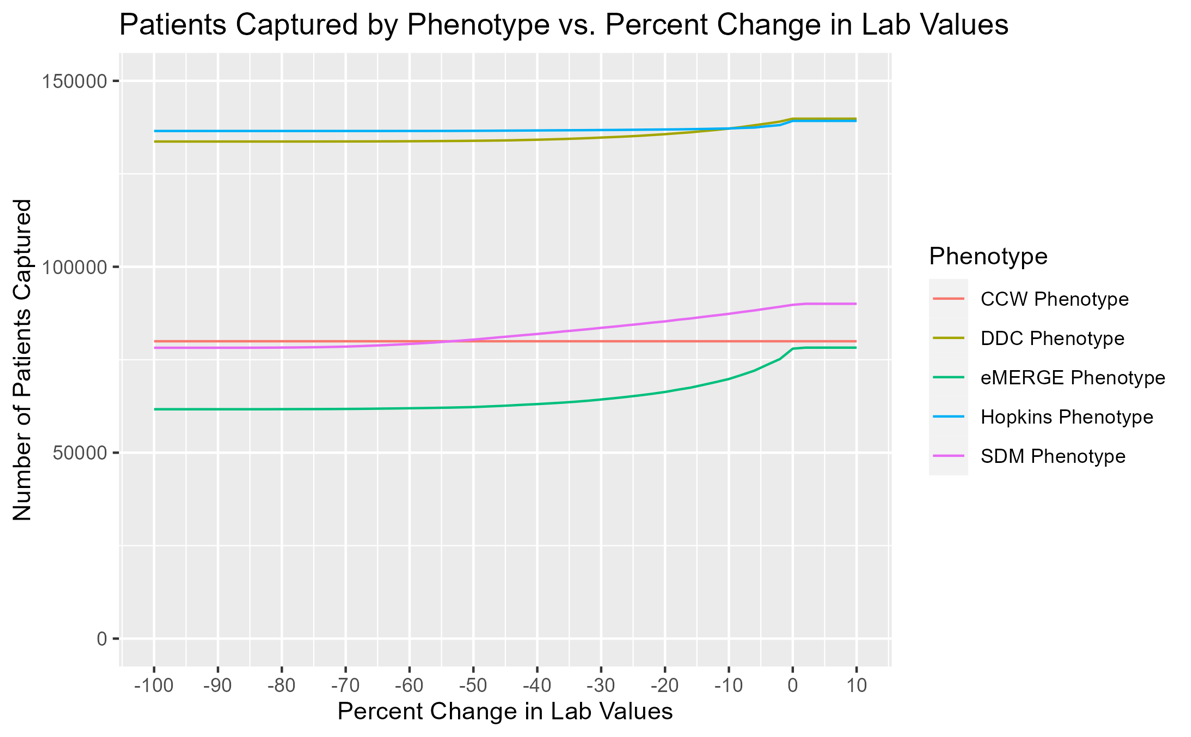


**Appendix Figure 12**. Number of T2D population identified by each T2D phenotype
definition with increasing inaccuracy of laboratory codes.

**
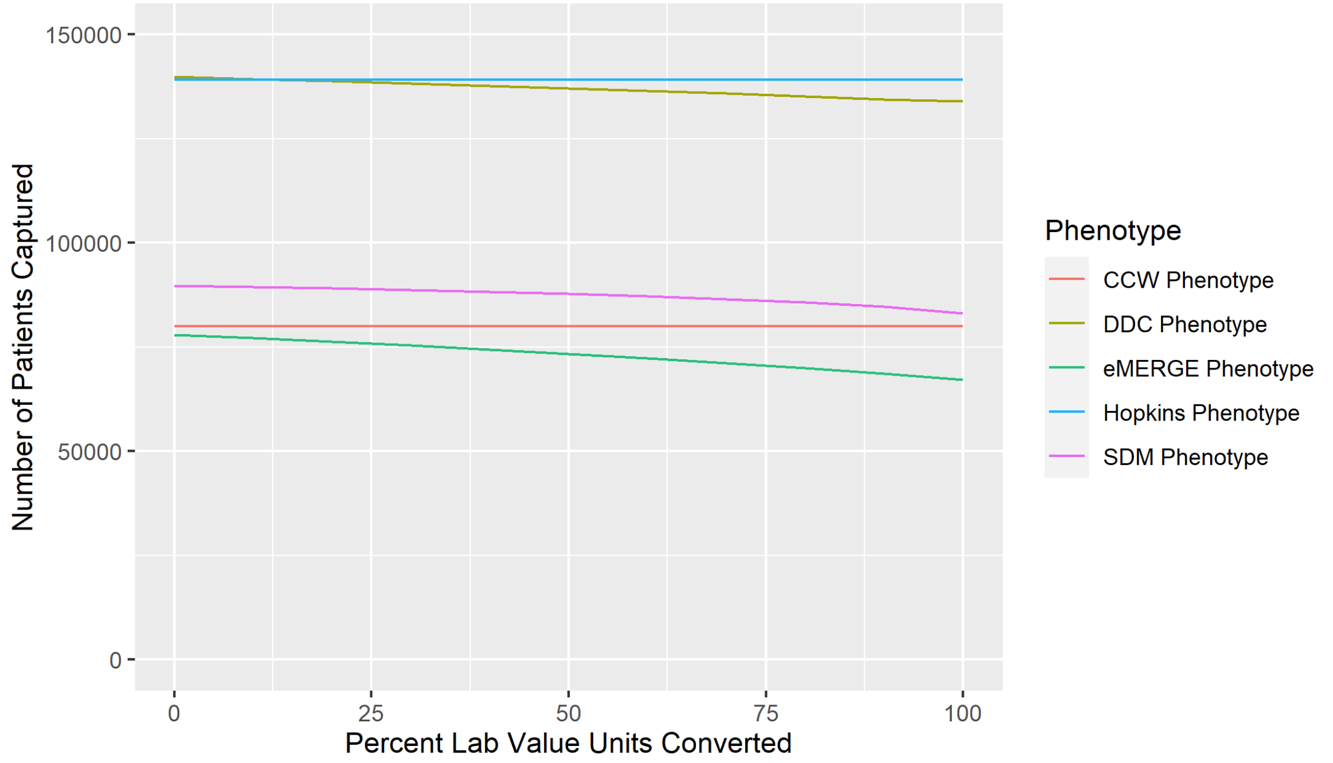
**

**Appendix Figure 13**. Number of T2D population identified by each T2D phenotype
definition with increasing inaccuracy of units of laboratory results.


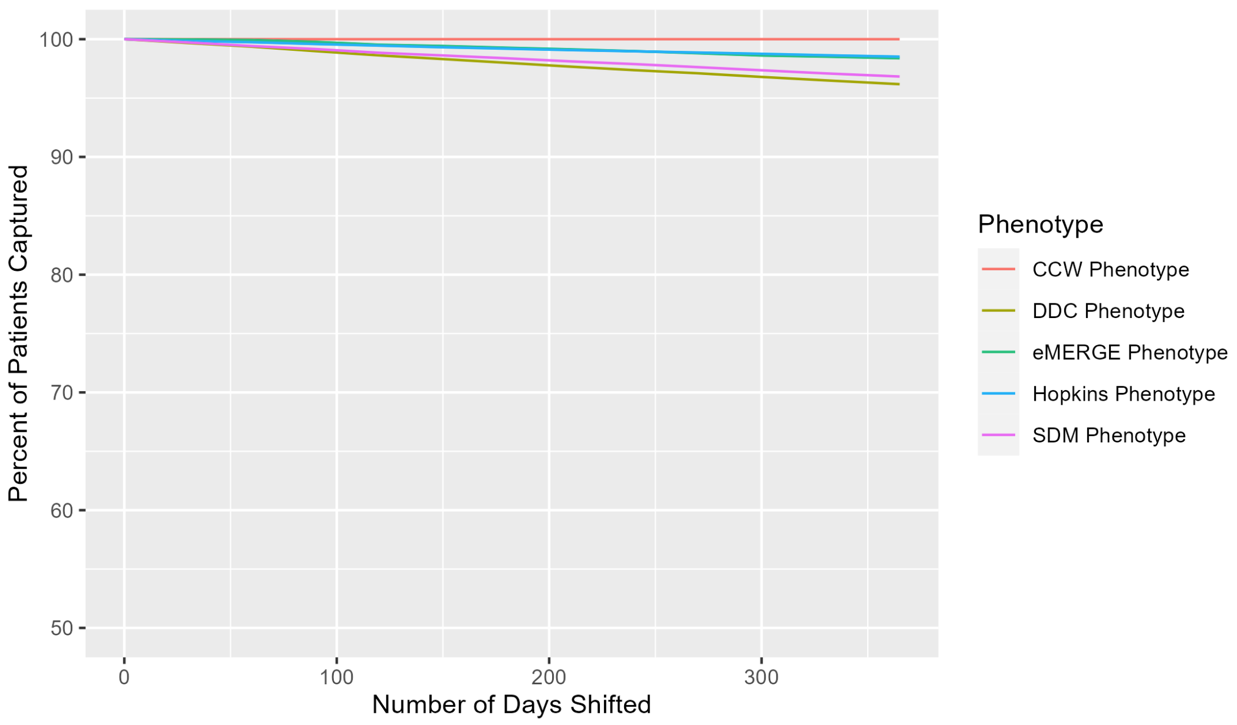


**Appendix Figure 14.** Percent of T2D population identified by each T2D phenotype definition
with increase shift in medication timeliness (i.e., days shift).


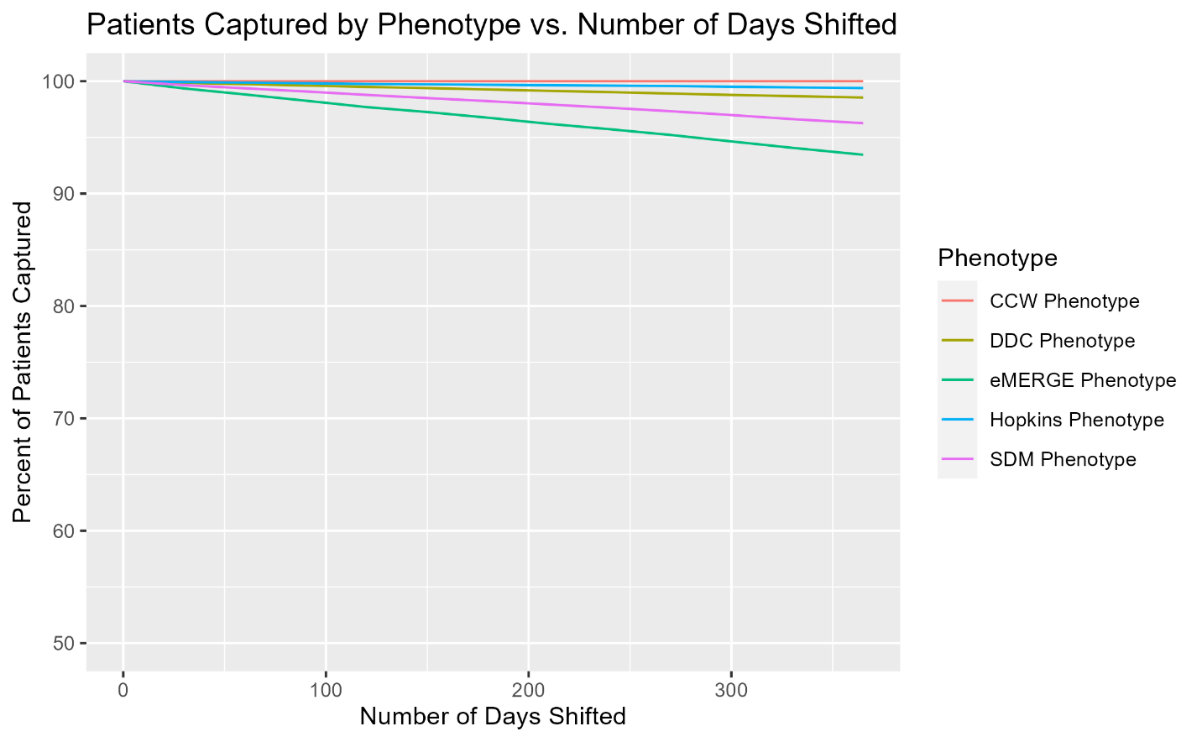


**Appendix Figure 15.** Percent of T2D population identified by each T2D phenotype definition
with increase shift in laboratory timeliness (i.e., days shift).


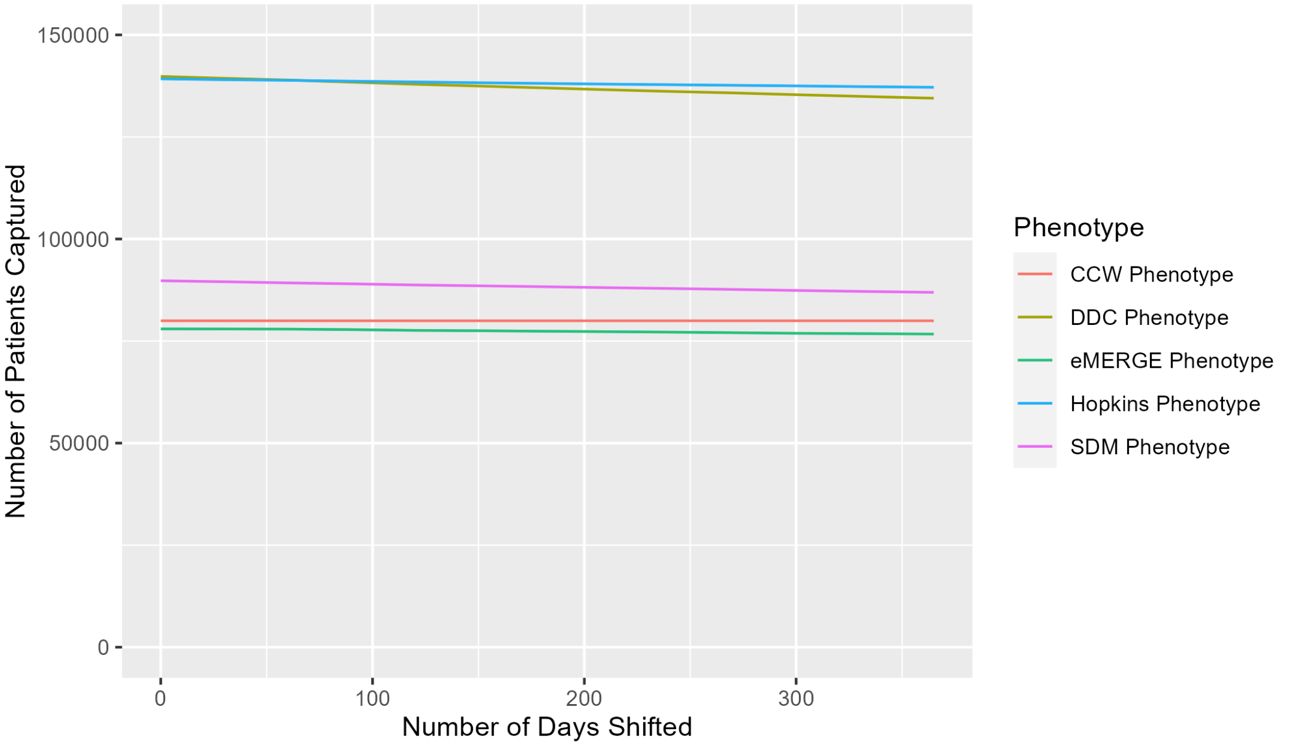


**Appendix Figure 16.** Number of T2D population identified by each T2D phenotype definition with
increasing shift in medication timeliness.


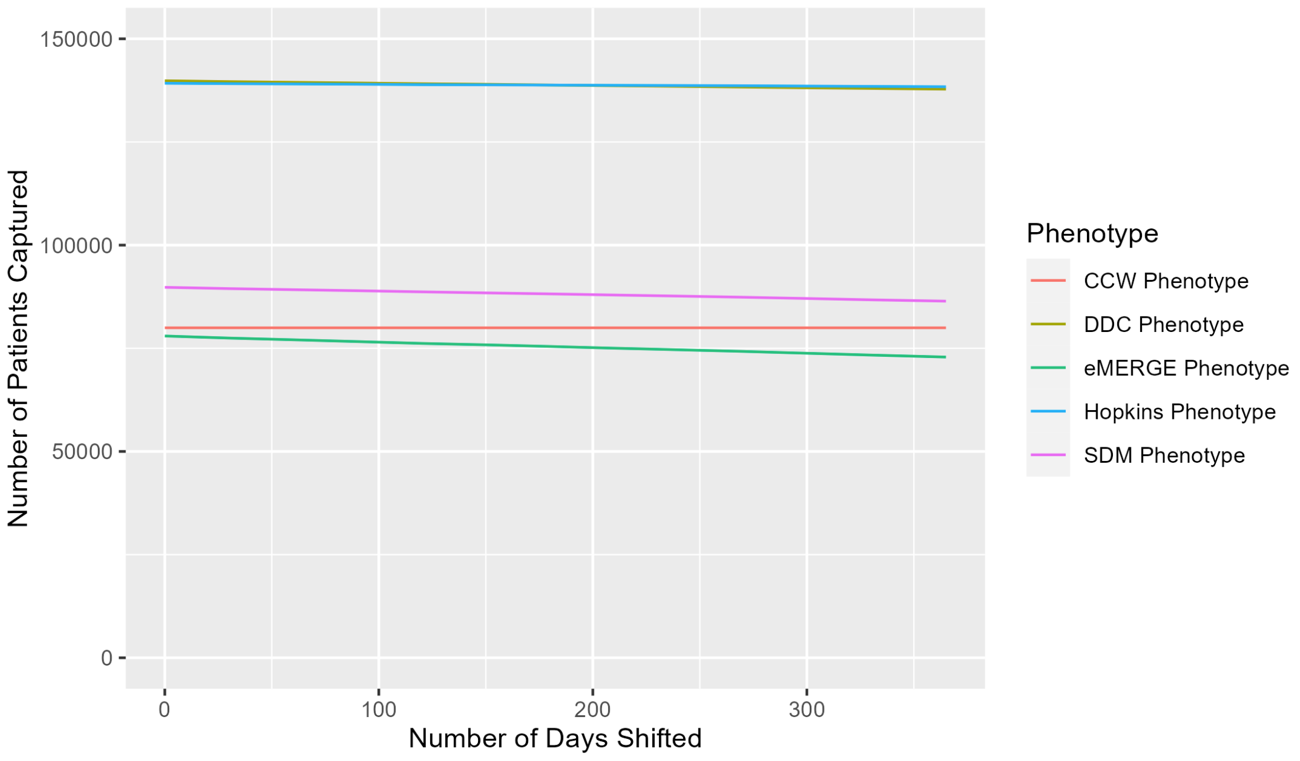


**Appendix Figure 17.** Number of T2D population identified by each T2D phenotype definition with
increasing shift in laboratory timeliness.


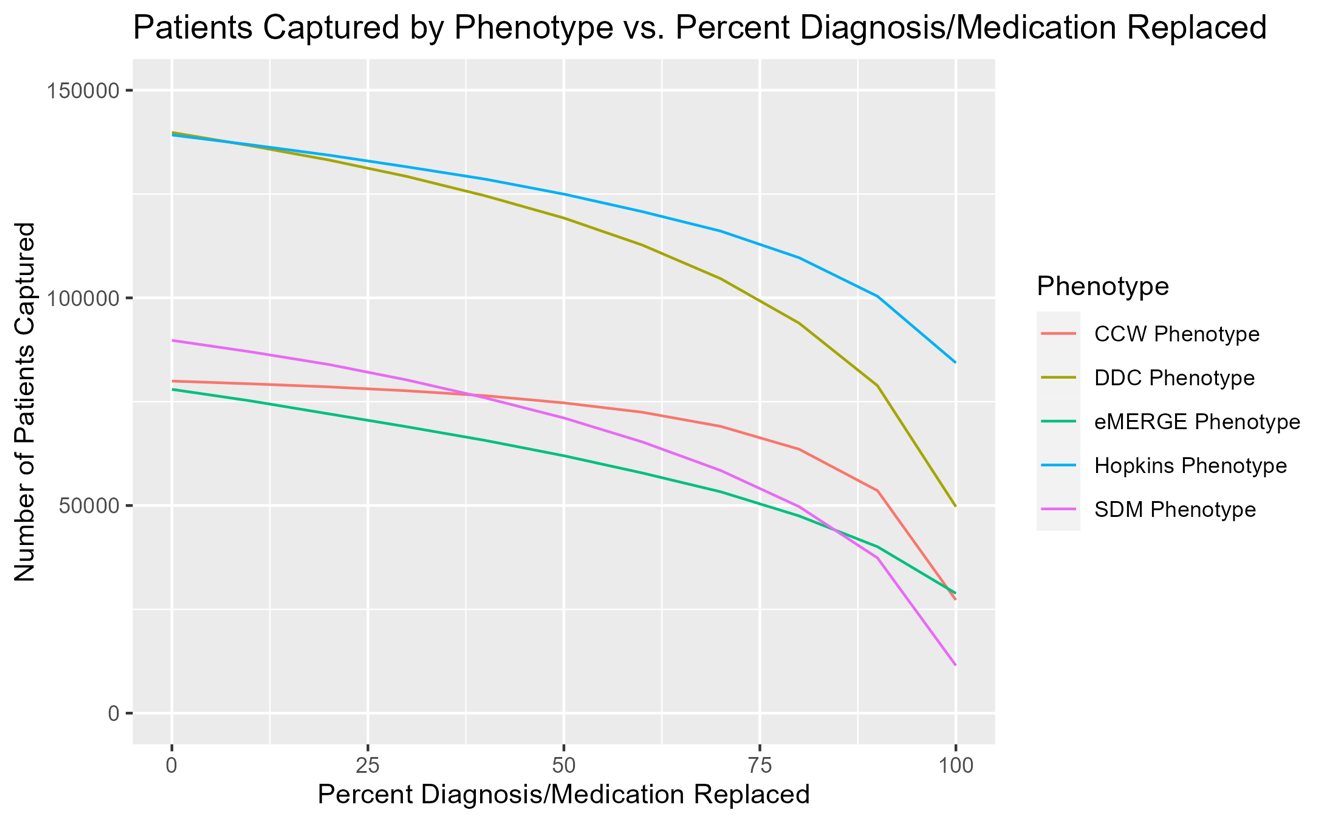


**Appendix Figure 18.** Number of T2D population identified by each T2D phenotype definition with
increasing replacement of both diagnostic and medication codes simultaneously.


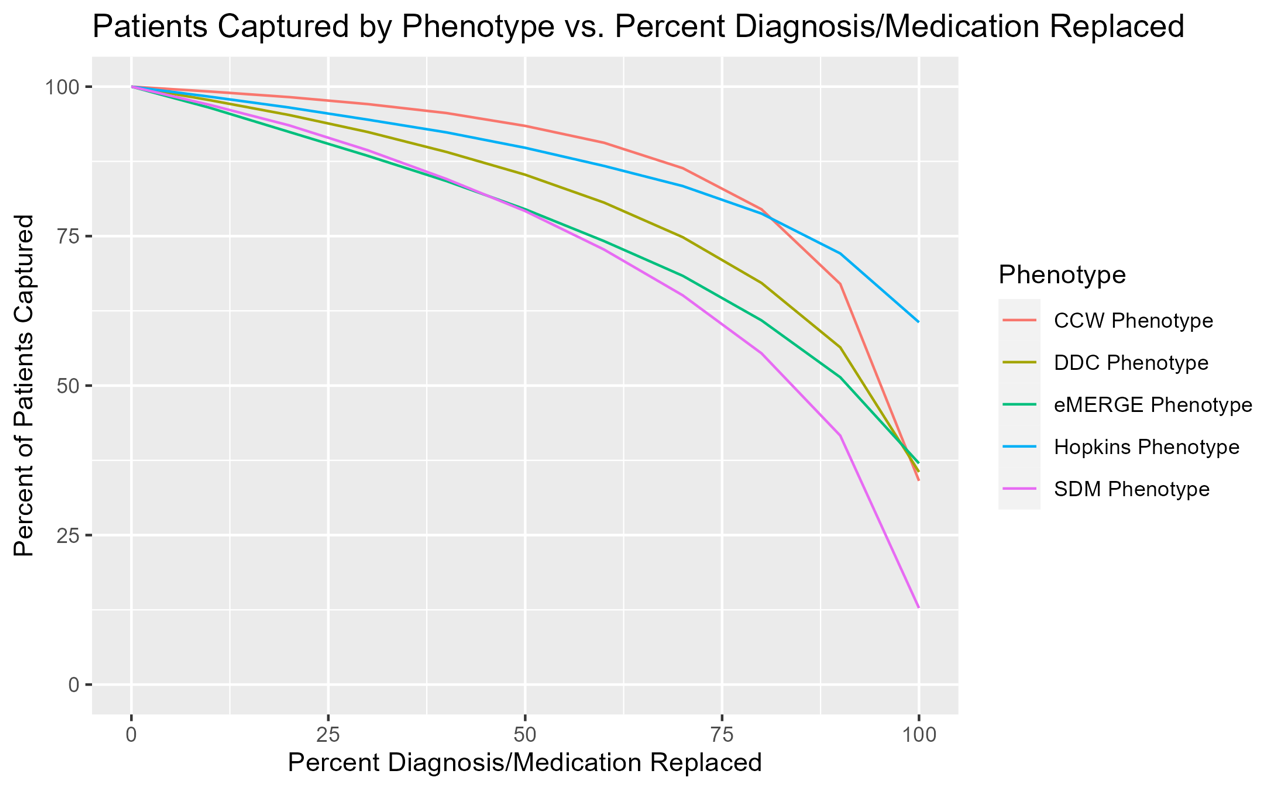


**Appendix Figure 19.** Percent of T2D population identified by each T2D phenotype definition
with increasing replacement of both diagnostic and medication codes simultaneously.


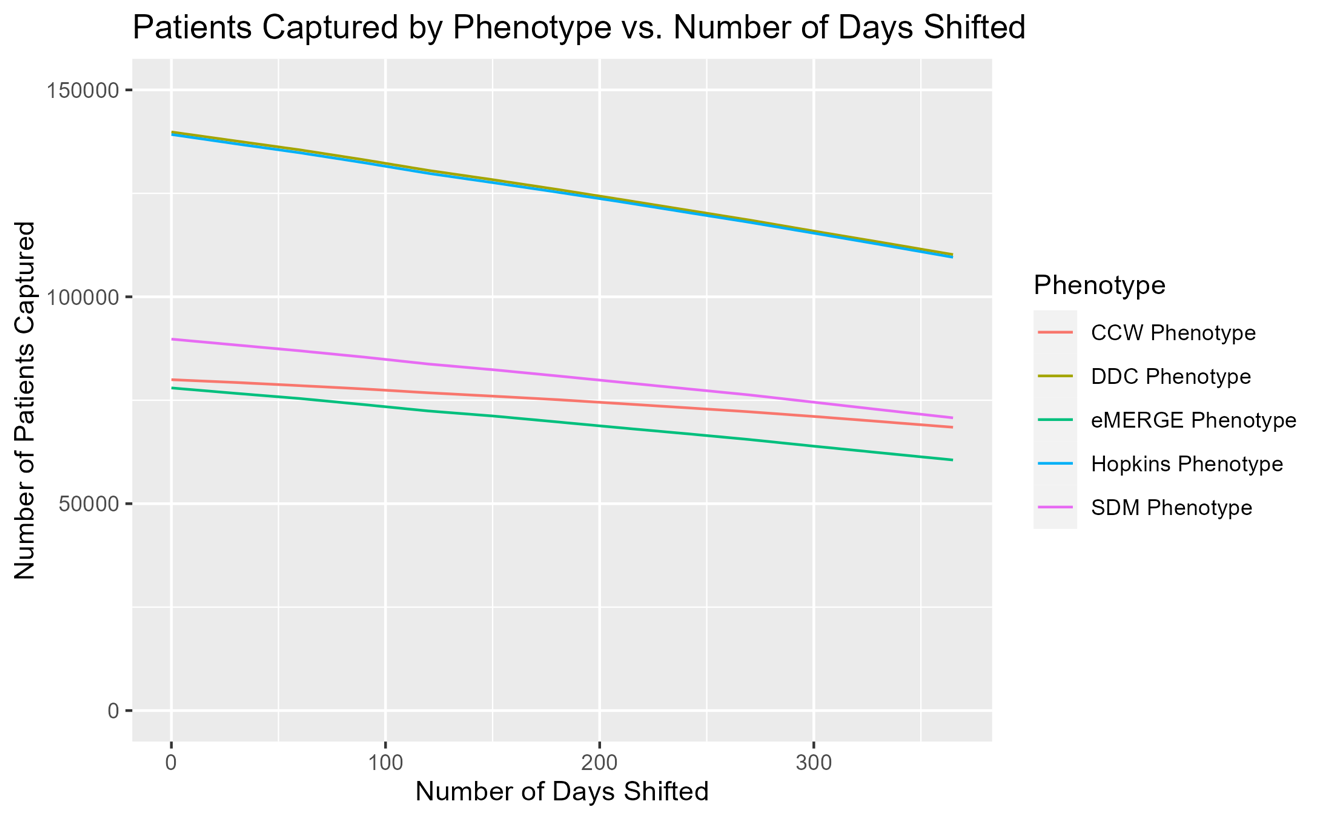


**Appendix Figure 20.** Number of T2D population identified by each T2D phenotype definition with
increasing date shifts in diagnostic, medication, and laboratory codes.


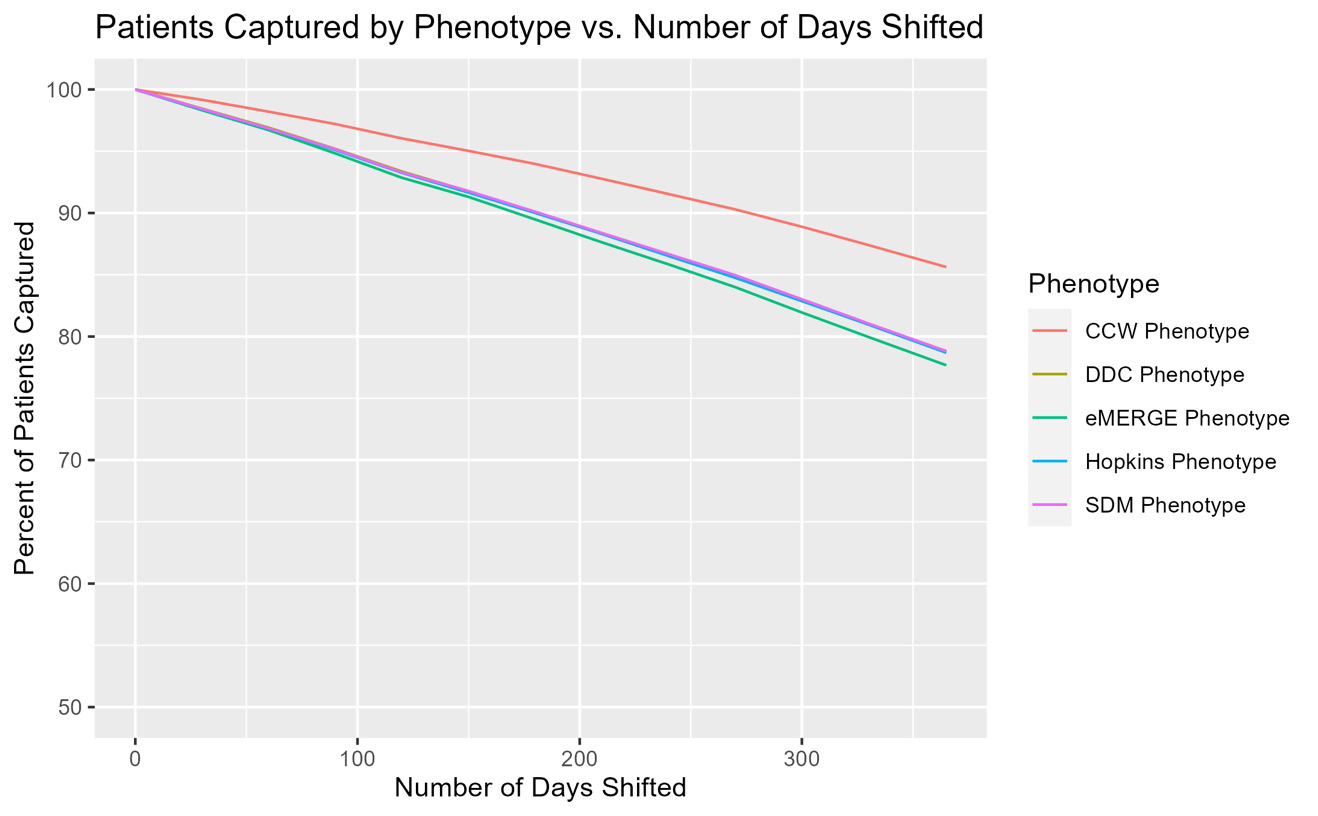


**Appendix Figure 21.** Percent of T2D population identified by each T2D phenotype definition with
increasing date shifts in diagnostic, medication, and laboratory codes.
